# Surfactant delivery in rat lungs: Comparing 3D geometrical simulation model with experimental instillation

**DOI:** 10.1371/journal.pcbi.1007408

**Published:** 2019-10-17

**Authors:** Alireza Kazemi, Bruno Louis, Daniel Isabey, Gary F. Nieman, Louis A. Gatto, Joshua Satalin, Sarah Baker, James B. Grotberg, Marcel Filoche

**Affiliations:** 1 Physique de la Matière Condensée, École Polytechnique, CNRS, Institut Polytechnique de Paris, Palaiseau, France; 2 ERL 7000 CNRS and IMRB U955 Inserm, Université Paris Est Créteil, Créteil, France; 3 Department of Surgery, Upstate Medical University, Syracuse, New York, United States of America; 4 Department of Biomedical Engineering, University of Michigan, Ann Arbor, Michigan, United States of America; University of California San Diego, UNITED STATES

## Abstract

Surfactant Replacement Therapy (SRT), which involves instillation of a liquid-surfactant mixture directly into the lung airway tree, is a major therapeutic treatment in neonatal patients with respiratory distress syndrome (RDS). This procedure has proved to be remarkably effective in premature newborns, inducing a five-fold decrease of mortality in the past 35 years. Disappointingly, its use in adults for treating acute respiratory distress syndrome (ARDS) experienced initial success followed by failures. Our recently developed numerical model has demonstrated that transition from success to failure of SRT in adults could, in fact, have a fluid mechanical origin that is potentially reversible. Here, we present the first numerical simulations of surfactant delivery into a realistic asymmetric conducting airway tree of the rat lung and compare them with experimental results. The roles of dose volume (V_D_), flow rate, and multiple aliquot delivery are investigated. We find that our simulations of surfactant delivery in rat lungs are in good agreement with our experimental data. In particular, we show that the monopodial architecture of the rat airway tree plays a major role in surfactant delivery, contributing to the poor homogeneity of the end distribution of surfactant. In addition, we observe that increasing V_D_ increases the amount of surfactant delivered to the acini after losing a portion to coating the involved airways, the coating cost volume, V_CC_. Finally, we quantitatively assess the improvement resulting from a multiple aliquot delivery, a method sometimes employed clinically, and find that a much larger fraction of surfactant reaches the alveolar regions in this case. This is the first direct qualitative and quantitative comparison of our numerical model with experimental studies, which enhances our previous predictions in adults and neonates while providing a tool for predicting, engineering, and optimizing patient-specific surfactant delivery in complex situations.

## Author summary

Surfactant Replacement Therapy (SRT), which involves instillation of a liquid-surfactant mixture directly into the lung airway tree, is a major therapeutic treatment in neonatal patients with respiratory distress syndrome (RDS). This procedure has contributed to a major decrease of the infant mortality in the past 35 years. Disappointingly, its use in adults for treating acute respiratory distress syndrome (ARDS) experienced initial success followed by failures. In this article, we present the first numerical simulations of surfactant delivery into realistic models of the conducting airway tree of the rat lung and compare them with experimental results. In particular, we show that the monopodial architecture of the rat airway tree plays a major role in surfactant delivery, contributing to the poor homogeneity of the end distribution of surfactant. This is the first direct qualitative and quantitative comparison of our numerical model with experimental studies, which enhances our previous predictions in adults and neonates while providing a tool for predicting, engineering, and optimizing patient-specific surfactant delivery in complex situations.

## Introduction

The alveolar surface of the lung is coated with a thin film of liquid [1]. In adult humans its surface area is ~90 m^2^ involving ~300–500x10^6^ alveoli whose average radius is ~100 μm. The pressure jump across the air–liquid interface tends to collapse them, unless the surface tension is significantly reduced. A reduced surface tension is also needed to increase the compliance, allowing the lung to inflate much more easily. This role is devoted to pulmonary surfactant, a complex mixture of lipids and proteins accumulating at the air–liquid interface [2]. Lack of surfactant can induce airway collapse (atelectasis) and low lung compliance. Its existence was first hypothesized by Von Neergaard who discovered that a larger pressure was needed to fill the lungs with air compared to saline [3]. In the 1950s, Pattle and Clements described the properties and functions of pulmonary surfactant [4–6].

Neonatal lungs have an alveolar surface area of ~3 m^2^ for ~24x10^6^ alveoli [7] whose average radius is about 75 μm, but can be as small as 40 μm in the case of premature neonates [8]. Prematurely born neonates can have a primary surfactant deficiency, which stiffens their lungs due to the high surface tensions, making them difficult to inflate. By studying the lung of infants who died from this neonatal respiratory distress syndrome (RDS), Avery and Mead inferred that the cause of death was due to surfactant deficiency from prematurity [9]. In the 1980s, after Fujiwara *et al*. reported the first successful trial of surfactant replacement therapy (SRT) for treating RDS [10], SRT became a standard therapy for these newborn RDS patients, contributing to a drastic drop in premature newborn mortality in less than 30 years (from 4,997 deaths in the US in 1980 to 861 in 2005 [11,12]). The dose volume, V_D_, per kilogram used successfully in neonates depends on the surfactant concentration. Examples are Survanta (25 mg/mL × 4 mL/kg = 100 mg/kg), Infasurf (35 mg/mL × 3 mL/kg = 105 mg/kg), and Curosurf (80 mg/mL × 1.25 mL/kg = 100 mg/kg) [13]. The move to smaller V_D_/kg, from 4 to 1.25 ml/kg, was to reduce the hazards of excess liquid in the lung while maintaining the molecular dose ~100 mg/kg, a goal based on a well-mixed compartment assumption.

SRT has also been proposed to treat a related condition affecting both children and adults called acute respiratory distress syndrome (ARDS), described for the first time by Ashbaugh *et al*. in 1967. ARDS is a generalized inflammatory process that causes the alveoli to flood with protein–rich fluid. It results from a variety of lung injuries such as pneumonia, sepsis, aspiration, trauma, and burns. It is characterized by an “acute onset of tachypnoea, hypoxemia” [14]. While not a primary surfactant deficiency as in neonatal RDS, the surfactant system in ARDS is severely compromised. Approximately 190,600 cases of ARDS in adults occur in the US every year with 39% mortality, i.e., 74,500 deaths [15]. SRT was initially successful in ARDS in adult patients [16], an ARDS model for large sheep [17], and later in pediatric patients to age 21 [18]. Common to these studies was a dose volume per kg range V_D_/kg = 2–4 mL.kg^-1^. The field, however, switched to a lower dose volume, V_D_/kg = 1–1.3 mL.kg^-1^, following a higher concentration strategy that was successful in premature neonates discussed above. However, it led to failure in adults [19–23].

In parallel to clinical studies, several groups performed careful experimental studies on liquid plug propagation in the pulmonary airway system. In 1994, Ueda *et al*. investigated surfactant–deficient ventilated preterm lambs [24], showing that surfactant distributions strongly depend on the chosen instillation technique/conditions. In 1998, Espinosa *et al*. showed that rapid injections lead to a more homogenous distribution [25]. Examining the effect of dose and delivery methods on 43–kg adult sheep, Lewis *et al*. observed in all cases an improvement of gas exchange: the larger the dose, the larger the improvement [17]. Moreover, tracheal instillation or administration directly into each lobe under bronchoscopic guidance showed similar results in terms of lobar distribution of surfactant. Cassidy *et al*. working on rat lung found that the formation of a liquid plug in the trachea before inspiration plays an important and positive role by creating a more uniform liquid distribution of surfactant throughout the lung [26]. In 2004, using radiographic image techniques, Bull *et al*. confirmed that the formation of liquid plugs in the large airways, which depend on posture and infusion rate, could result in a more homogeneous liquid distribution than gravity drainage alone [27]. Finally, in 2017, Steffen *et al*. showed that SRT was able to lower alveolar surface tension and that the number of open alveoli was improved significantly in a rat model [28].

Theoretical studies on liquid/surfactant delivery to the lung have also been performed. Halpern *et al*. discovered that both transit and delivery times are strongly influenced by the amount of pre–existing surfactant and by coating the conducting airway surface area treated as a trumpet model [29]. Mathematical modeling and numerical simulations have been used to assess SRT efficacy, which is not only determined by the amount of surfactant delivered to the targeted (alveolar) regions, but also by the homogeneity of the end distribution of delivered surfactant. This distribution in the alveolar region is affected by a number of factors, including physical properties of the liquid (viscosity, density, surface tension), patient posture (prone/supine, left lateral decubitus/right lateral decubitus) airway geometry, instillation method (flow rate), and presence of other plugs in nearby airways from previous instillations [30].

In 2011 and 2015, we published the first 3D structural model of SRT, simulating the delivery of a liquid bolus of surfactant mixture into the entire tracheobronchial tree [31,32]. The model is a two–step process, first of a liquid plug propagating down an airway tube forced by airflow, and then plug splitting at an airway bifurcation. These two steps are then repeated in the daughter airways and so on through the airway tree. Airway coating occurs during the first step, which reduces the amount available to the acinus. At the bifurcation the effects of gravity and branching asymmetry determine the split ratio of volumes going into each daughter. Using this approach, we showed that during SRT a fraction of V_D_ is lost coating the airways, called the *coating cost* V_CC_. So, the amount actually reaching the acinus is V_D_–V_CC_. SRT remains clinically effective in neonates even at low values of V_D_/kg thanks to the large amount V_D_–V_CC_, in stark contrast with adults in which large values of V_D_/kg are required due to the high coating cost [31]. The adult lung is not a well–mixed compartment for SRT. The major difference influencing V_D_–V_CC_ in neonates compared to adults is the airway surface area available for V_CC_. In a Weibel’s model of the lung, the airway surface area of a neonate, which has ~8 conducting airway generations, is ~40 cm^2^ while that of an adult, with ~15 generations, is ~4,500 cm^2^. This is a ~110:1 ratio between adult and neonate surfaces areas, a quantity that does not scale with weight since it significantly exceeds the kg weight ratio of ~70:1–3 adult to neonate in this patient population [31–33]. An interesting feature of the model is that increasing flow rate makes for a thicker coating layer, so increases V_CC_ and reduces delivery, but makes the splitting more even, so improves the homogeneity of the final acinar distribution.

The present study investigates the propagation of surfactant into rat lungs. To that end, we have first developed a realistic 3D geometrical model of the rat conducting airways based on morphometric measurements. Simulations of surfactant delivery have been carried out using the model introduced in [31–33]. The respective effects of lung asymmetry, dose volumes, flow rates, and repetitive dosing are studied. The numerical results show very good agreement with experimental data and support our model of surfactant delivery.

## Materials and methods

### Geometrical model of the rat lung

**Fig 1:**
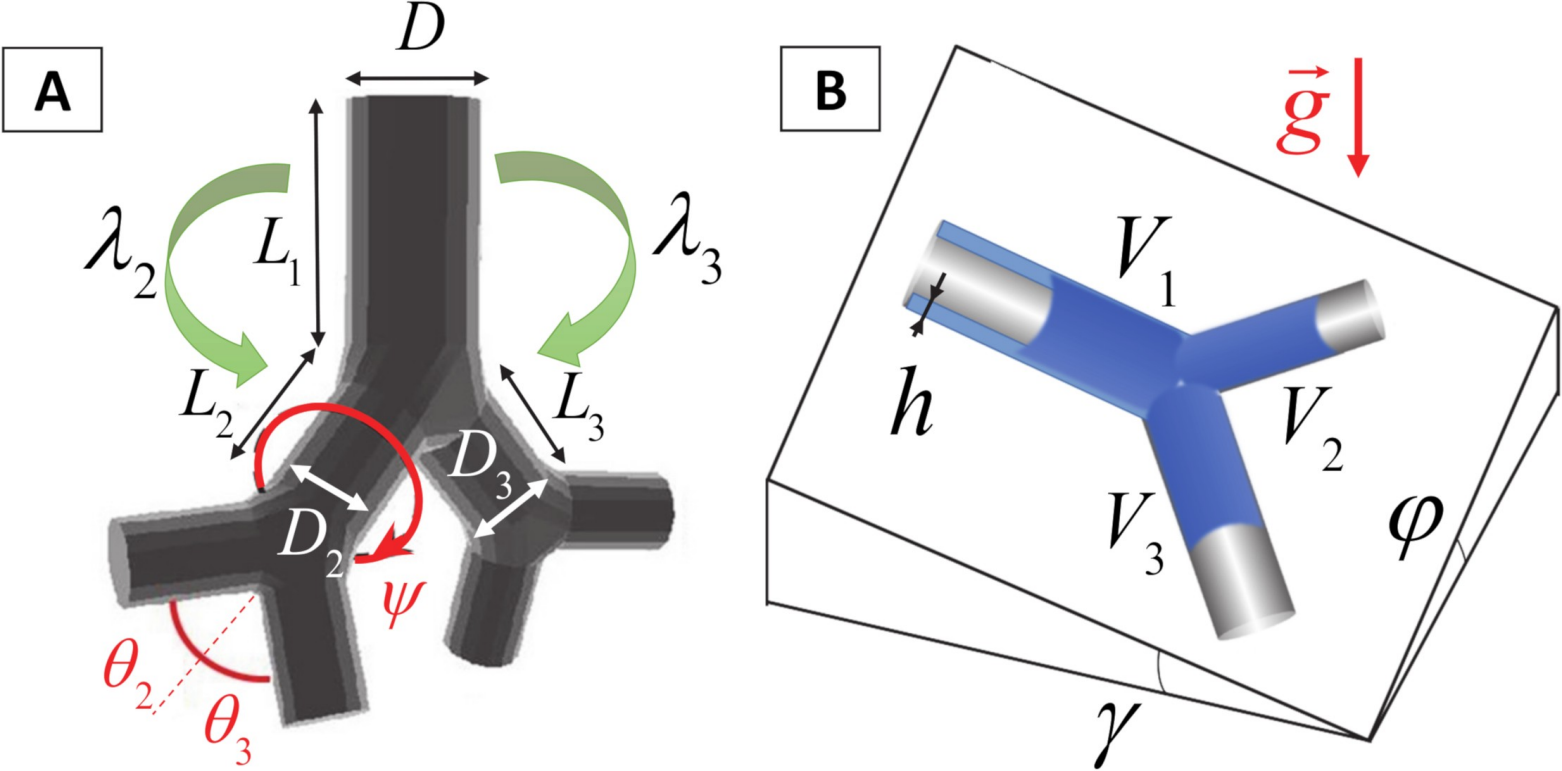
Geometric description of the airway tree. A) The airways are described by their diameter (*D*) and length (*L*). A single bifurcation is characterized by its branching angles (*θ*_2_ and *θ*_3_) and by its diameter ratios (*λ*_*i*_ = *D*_*i*_/*D*_1_, *i* = 2,3). The relation between consecutive bifurcations is characterized by the rotation angle between their respective bifurcation planes (*ψ*). B) Flow across one single bifurcation: the spatial orientation of the bifurcation is characterized by two angles: the pitch angle (*γ*, which is the angle to gravity of the parent branch), and the rolling angle (*φ*, which the angle to gravity in the perpendicular direction). A plug of volume *V*_1_ entering the parent branch leaves behind a trailing film of thickness h. The volumes entering the daughter branches are *V*_2_ and *V*_3_ such that *V*_2_+*V*_3_<*V*_1_.

**Fig 2:**
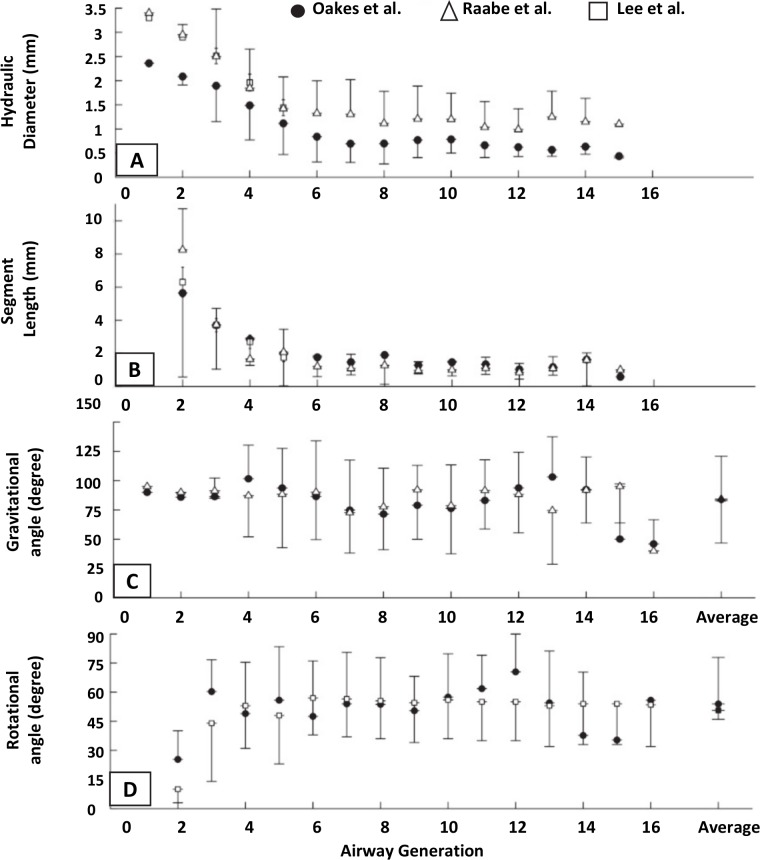
Comparison between measurements of Oakes *et al*. performed on Wistar rats [36], measurements of Raabe *et al*. performed on Long-Evans rats [35], and measurements of Lee *et al*. performed on Sprague-Dawley rats [67].

**Fig 3:**
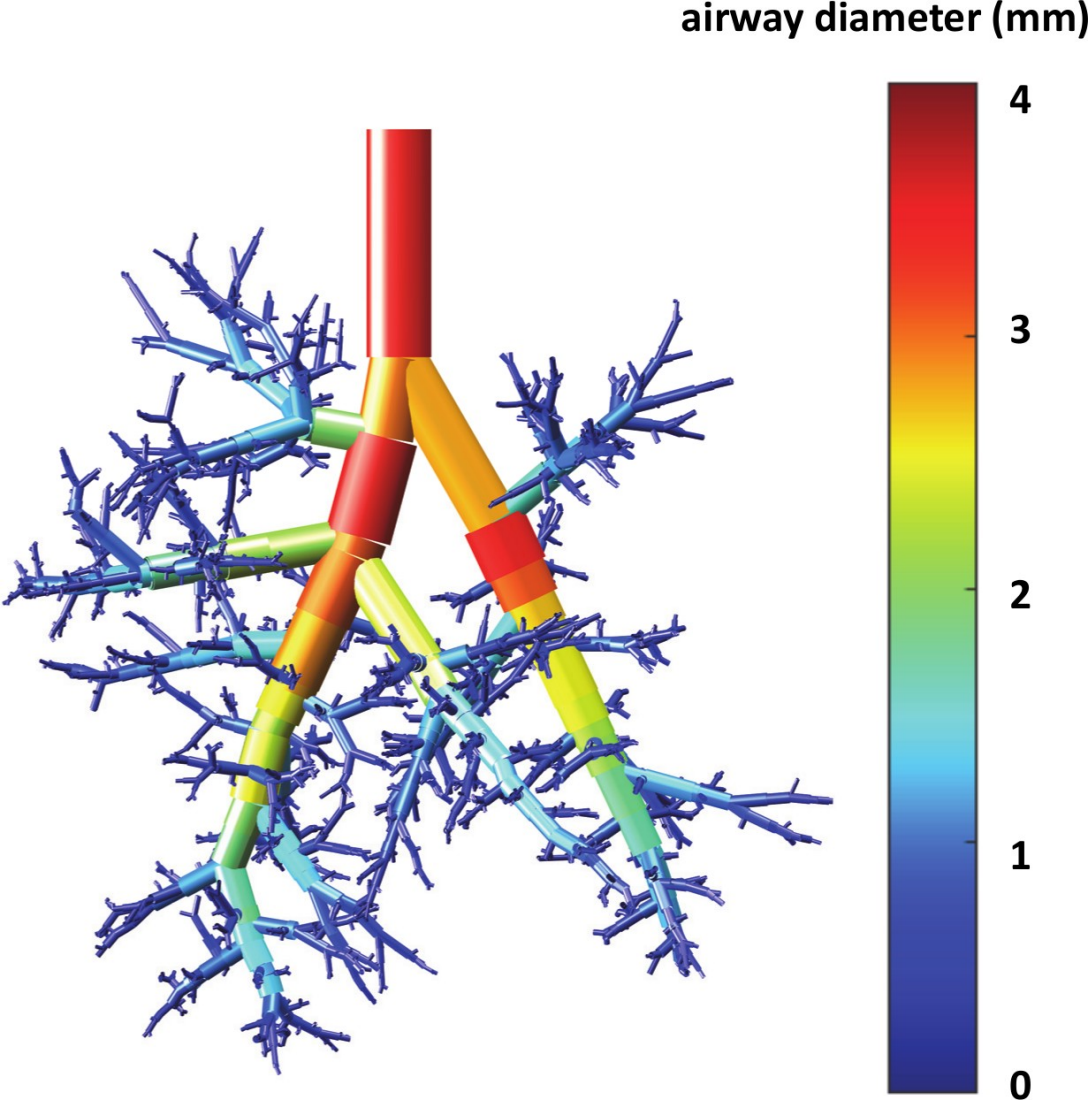
0.33-kg rat lung airway tree (30 generations). The color-coding corresponds to the value of the diameter.

**Fig 4:**
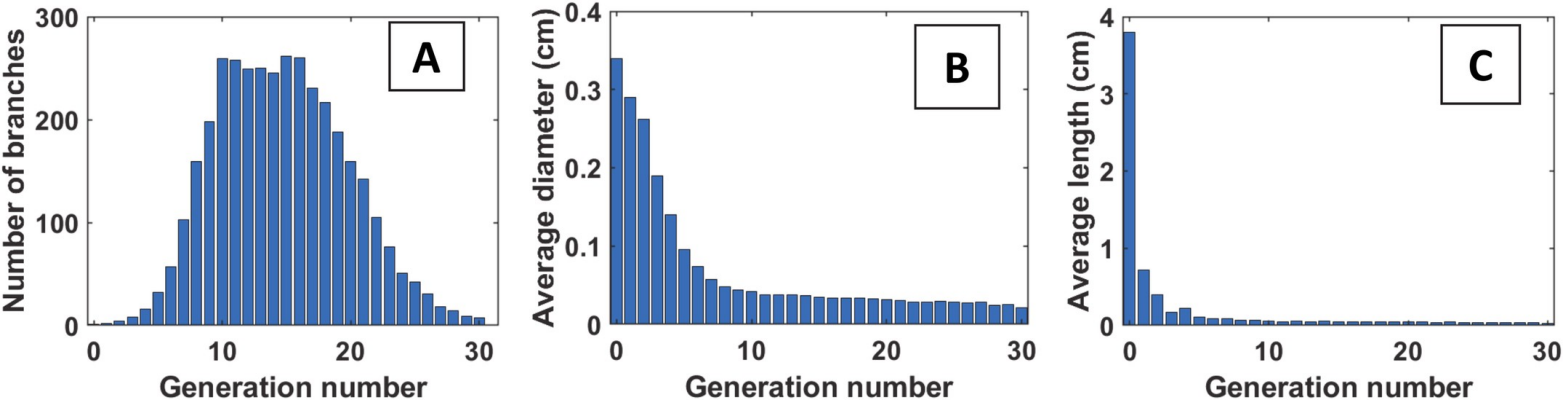
Statistics of the rat lung model built from [35]. A) Number of branches vs. generation. B) Average diameter vs. generation. C) Average length vs. generation.

The lower respiratory tract of the rat is organized as an asymmetrically branching tree. The mature rat lung has an alveolar surface area of 0.8 m^2^ involving 82×10^6^ alveoli [34] whose average volume is 12×10^4^ μm^3^, corresponding to an average radius of 30 μm in a spherical approximation. We have developed a geometrical model of this tree in which the rat pulmonary airway system is described as an assembly of connected straight tubes defined by their diameters (*D*), lengths (*L*), branching angles (*θ*), and rotation angles between successive bifurcation planes (*ψ*) (see Fig 1). The values of these parameters are obtained from morphometric measurements of Raabe *et al*. performed on flexible silicone rubber replica casts of Long-Evans rats [35] at a volume corresponding to end inspiration. Although Wistar and Sprague-Dawley rats were used in the experimental studies used for our comparisons, we chose to generate and simulate Long-Evans rats due to the extensive database on their lung structure (branching angles), and the relative similarity between Long-Evans, Wistar, and Sprague-Dawley lung geometries. In 2012, Oakes *et al*. [36] studied statistics of the branching pattern of Wistar rat. Fig 2 displays a comparison between average values of diameter, length, angle to gravity, and rotation angle vs. generation obtained from Oakes, Raabe, and Lee studies, respectively. This comparison shows that Wistar rat airway diameters are slightly smaller than Long-Evans and Sprague-Dawley for distal generations (Fig 2A), while lengths are essentially identical (Fig 2B). Table 1 shows a general comparison between several parameters of these trees, i.e., angles of airways to gravity, rotation angles, bifurcation angles, body weight, and lung volume. One can see the similarity between these trees. The Long-Evans tree used in our simulations starts from a 3.4 mm diameter trachea, contains at most 30 generations along the longest path, and has 1457 terminal branches (see Fig 3). Fig 4 displays the number of branches at each generation as well as the average diameter and length. The airways are classified by generations, labeling the trachea 0 and increasing by 1 at each bifurcation following Weibel’s definition [37].

**Table 1:** Compared lung geometry between Wistar, Long-Evans, and Sprague-Dawley rats.

	Raabe 1976 [35]	Oakes 2012 [36]	Lee 2008 [67]
**Species**	Long-Evans	Wistar	Sprague Dawley
**Diameter**	Hydraulic diameters measured in Long-Evans and Sprague-Dawley rats are very similar (see Fig 2). Wistar rats exhibit systematically smaller diameters.		
**Length**	Airway lengths in all three databases are very similar (see Fig 2).		
**Bifurcation Angle**	19.3° ± 14.6° for the major airway in the Wistar rat, i.e. 36.7% larger than in the Long-Evans rat, 60.5° ± 19.4° for the minor airway in the Wistar rat, i.e. 4.6% smaller than in the Long-Evans rat		
**Angles of airways to gravity**	35°-85°	38°-83°	-
**Rotation angles**	-	24–53	20–50
**Body Weight**	330 g	268 g	302 g
**Fraction of Total Lung Volume, %**			
**Right apical**	10.4	11.0 ± 1.16	9.2
**Right diaphragmatic**	28.8	28.0 ± 1.02	31.8
**Right intermediate**	13.9	13.5 ± 0.36	13.3
**Right cardiac**	12.1	11.6 ± 0.51	12.5
**Left lung**	34.8	35.9 ± 1.3	33.3
**Right lung (all lobes)**	65.2	64.1 ± 1.3	66.8

### Model of propagation of a liquid plug into the airway tree

The tracheobronchial trees simulated in our model are considered as solid bodies whose geometry does not depend on the tree orientation. We thus do not account for the elastic properties of the lung that may change the volume and shape of the airway tree during exhalation and inhalation, or when assuming different rat postures. When surfactant is instilled in sufficient amount into the pulmonary airway tree, a liquid plug forms that propagates along the branches of the pulmonary airway system towards the acinar region. In each airway, the liquid plug loses a fraction of its volume left coating the airways wall. At the end of the airway, the plug divides to enter the two daughter airways. Following the mathematical model introduced in [30,31,38], the intricate dynamics of the liquid plug during its propagation into the tree structure is thus simplified into two separate fundamental steps: step A, steady propagation of the plug along an individual airway and deposition of a trailing film onto the airway walls; step B, plug splitting at the bifurcation. As shown in [31], step A essentially governs the efficiency of the delivery, defined as the fraction of the initial plug volume reaching the terminal region, while step B mostly governs the homogeneity of the final distribution.

The trailing film thickness in step A is determined using a phenomenological equation derived in [39]:

$$H=\frac{h}{a}=0.36\left(1-e^{-2Ca^{0.523}}\right),$$ (1)

where *h* is the trailing film thickness, *a* is the airway radius, and *Ca* is the capillary number (*Ca* = *μU*/*σ*) that represents the ratio of viscous forces to surface tension force (*μ*: viscosity, *U*: velocity, *σ*: surface tension). Computational studies carried out by Heil [40] showed less than 10% variation of H over a Reynolds number range 0<Re<300. The range of Re in the rat simulations is 0–200. Consequently, ignoring inertia for the trailing film thickness in Eq (2) is reasonable.

Step B is characterized by a quantity called the *splitting ratio*, *R*_*s*_, defined as the ratio of the volume of liquid entering the upper daughter, *V*_2_, to the volume entering the lower daughter, *V*_3_: *R*_*S*_ = *V*_2_/*V*_3_ (see Fig 1). A value of *R*_*S*_ = 1 means that the plug splits equally while *R*_*S*_ = 0 or *R*_*S*_ = +∞ means that the entire plug flows down into one of the daughter airways. For practical reasons and to avoid handling quantities that may become infinite, we choose to use, instead of *R*_*s*_, a *splitting factor α* which is a dimensionless number comprised between 0 and 1 defined as:

$$\alpha =\frac{R_{s}}{R_{s}+1},$$ (2)

so that *V*_2_ = *αV* and *V*_3_ = (1−*α*)*V*. A value *α* = 0.5 means that the plug splits equally between both daughter airways while values 0 and 1 correspond a fully uneven splitting.

Using conservation of momentum and mass, and assuming Poiseuille flow inside the airways, we derive a quadratic equation in *R*_s_ (see Supporting Information):

$$A\alpha^{2}+B\alpha +C=0,$$ (3)

where *A*, *B*, and *C* can be expressed as functions of 3 dimensionless parameters: the Reynolds number (Re = *ρUa*/*μ*) which represents the ratio of inertial to viscous forces, the Bond number (Bo = *ρga*^2^/*σ*) which represents the ratio of gravitational forces to surface tension force, and the aforementioned capillary number. The three constants *A*, *B*, and *C* read

{A=ReCa(1λ24−1λ34)+16V˜1Ca(1λ26−1λ36)B=2V˜1Bo(f2λ22+f3λ32)+2ReCaλ34+32V˜1Caλ36C=−ReCaλ34−16V˜1Caλ36−2V˜1Bof3λ32+4(1λ3−1λ2) (4)

where *λ*_*i*_ = *D*_*i*_/*D*_parent_ (*i* = 2,3) are the ratios of daughter to parent airway diameters, ${\tilde{V}}_{1}=V_{1}/\pi a^{3}$ is the dimensionless dose volume, *V*_*1*_ being the actual dose volume reaching the bifurcation, and *f*_*i*_ = sin *θ*_*i*_ sin *φ*−cos *θ*_*i*_ sin *γ*, in which *θ*_i_ (*i* = 2,3), *φ*, and *γ* are the half-branching angle, the roll angle, and the pitch angle, respectively [30,31,38] (see Fig 1).

If we assume a symmetric airway tree (*D*_2_ = *D*_3_, *L*_2_ = *L*_3_, *θ*_2_ = −*θ*_3_ = *θ*), then

α=12(1−Xsinθsinφ1−Xcosθsinγ)withX=2BoV˜1λ4(Reλ2+16V˜1)Ca (5)

Two key quantities dominate plug splitting at each bifurcation: plug velocity and gravity orientation. A higher velocity tends to even the splitting while on the contrary gravity tends to favor the downwards oriented airway, making the split more asymmetric. In a normally breathing rat, with a flow rate of about 30 mL.kg^-1^.s^-1^ in the trachea, typical values of the dimensionless parameters Re and Ca are ~61and ~1. These values drop down to 0.06 and 0.014, respectively, in the terminal bronchioles, while their average values in the entire tree are 2 and 0.17, respectively. Bond number is about 1 in the trachea and 0.04 on average in the entire tree.

**Fig 5:**
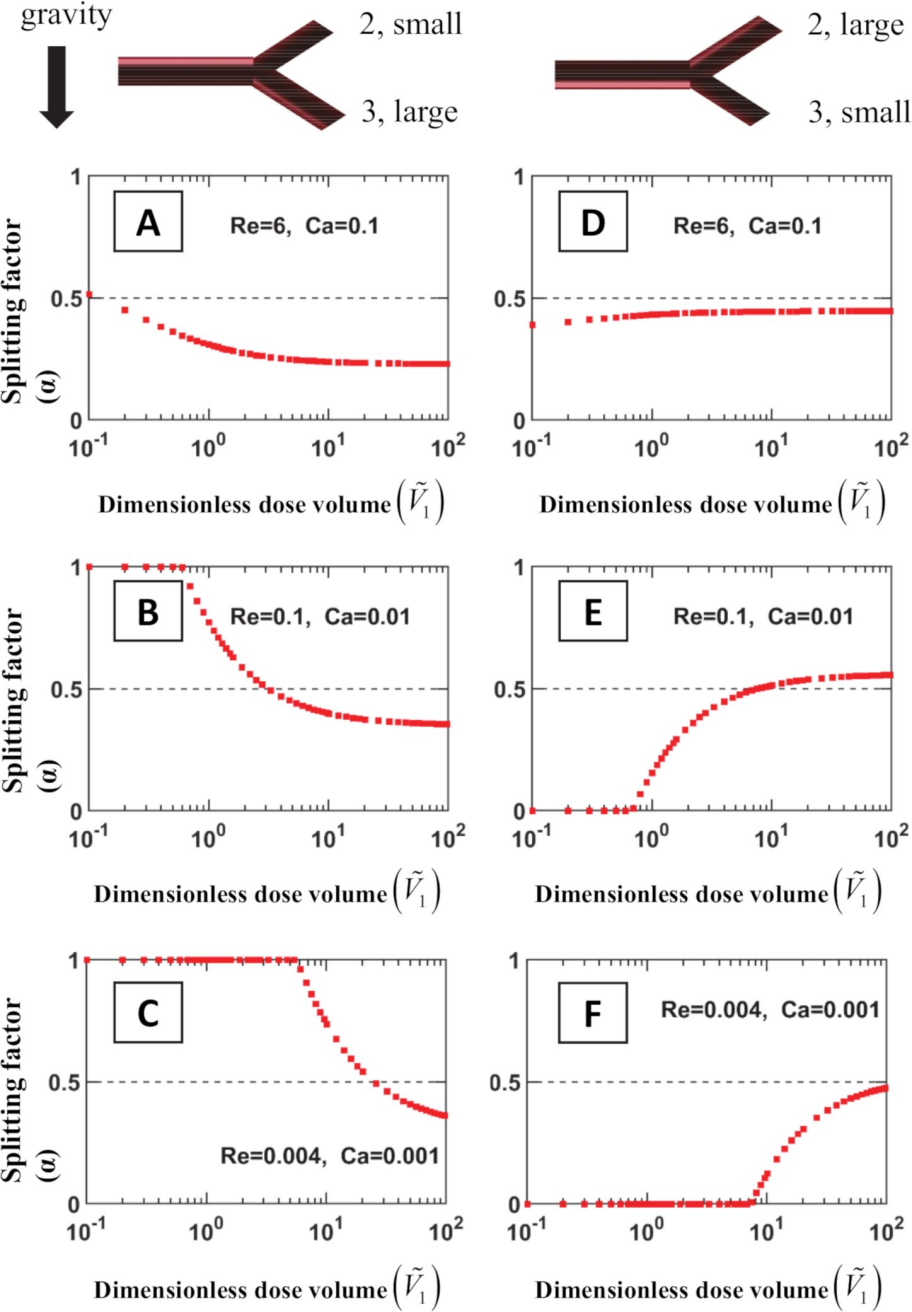
Splitting factor *α* = *V*_2_/(*V*_2_+*V*_3_) vs. dimensionless incoming volume ${\tilde{V}}_{1}$ for various values of Ca and Re, in a vertical asymmetric bifurcation. The branching angles are both 45°, and the diameter ratios of daughter to parent are 0.83 and 0.72, for the large and the small daughter airways, respectively. In the left column (frames A to C), the smaller airway goes up while the larger goes down. In the right column (frames D to F), the situation is reversed. Horizontally, the values of Re and Ca in frames A and D correspond to the trachea (for a delivery at a flow rate of 6 mL.kg^-1^.s^-1^); the values of Re and Ca in frames B and E correspond to an intermediate airway (generation 6), while frames C and F correspond to a distal airway. One can observe that at small values of ${\tilde{V}}_{1}$, tend to always favor the smaller airway independently of the orientation due to the predominance of capillary forces, while larger values of ${\tilde{V}}_{1}$ favor the larger airway. (Reducing the flow rate for a given airway however would always favor the downwards airway, as shown in **[38]**).

Besides the plug velocity, another parameter plays an important role in determining the fate of the plug after a bifurcation: the plug volume. Fig 5 displays how the dimensionless dose volume ${\tilde{V}}_{1}$ (the plug volume divided by π*a*_1_^3^) of a plug can modify its splitting factor *α*, for various values of the capillary and the Reynolds numbers in a vertical asymmetric bifurcation. The left column (frames A to C) corresponds to the situation where the larger daughter airway goes down while the smaller daughter airway goes up. The right column (frames D to F) presents the opposite situation in which the larger daughter airway goes up. Horizontally, frames A and D correspond to typical values of Ca and Re in the trachea, frames B and E to an intermediate airway, and finally frames C and F to a very distal airway. All cases are computed for a tracheal flow rate per kg of 6 mL.kg^-1^.s^-1^ which is similar to the experimental conditions presented later in the article. Our computations show a rather complicated picture: depending on the plug volume, a major fraction of the plug volume reaching the bifurcation can either go upwards or downwards (see Fig 5). For small values of ${\tilde{V}}_{1}$, one observes that most of the plug goes to the smaller daughter due to the predominance of surface tension effects. At larger values of ${\tilde{V}}_{1}$, on the contrary, the largest airway is favored.

### Modeling multiple aliquot instillation

**Fig 6:**
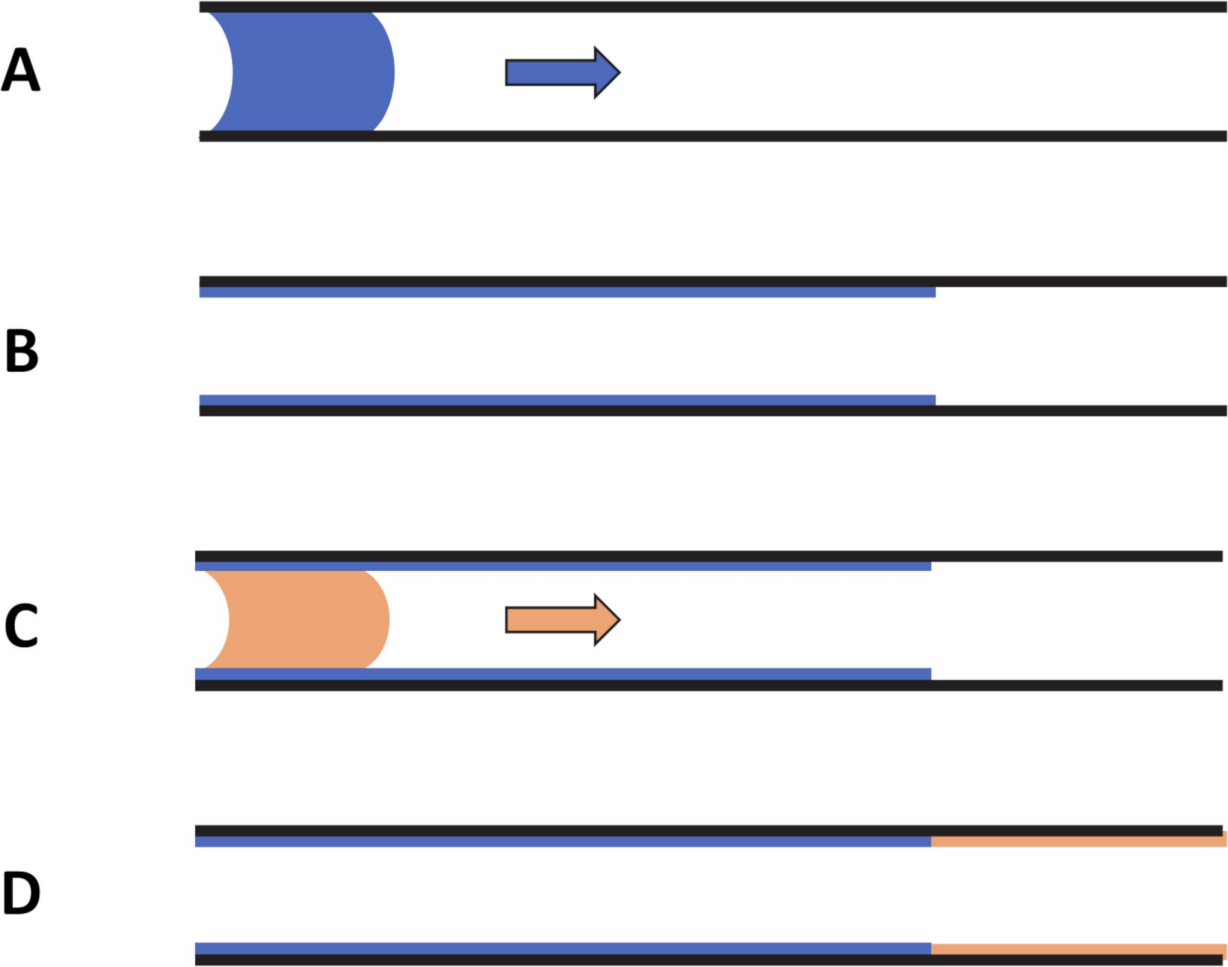
Sketch of the liquid plug model. A) and B) A liquid plug propagating in an airway with dry walls leaves a trailing film and may end up being entirely deposited on the airway wall. C) and D) A second plug (in orange) propagating into an airway whose walls are already coated with surfactant proceeds without losing its mass, and starts leaving a trailing film only when it reaches the dry portions of the walls.

Surfactant can be delivered into the lung either in one single or through multiple instillations. In the latter case, the initial dose volume is divided into equal aliquots, one per breath. When instilling the first aliquot, a plug forms which propagates along the branches of the pulmonary airway system. In each airway, the liquid plug loses a fraction of its volume that remains coating the airways walls (Fig 6A and 6B). The following aliquots form new plugs that therefore propagate through airways already lined with surfactant.

In a previous work of our team, Cassidy *et al*. [26] used a pre-wetted tube to assess the effect of a precursor (preexisting) film on the coating layer and plug length. We observed that the trailing film thickness depends upon the plug capillary number (Ca) but not on the precursor film thickness. In addition, we have analyzed steady liquid plug propagation in a two-dimensional channel using computational fluid dynamics [41,42]. The flow is steady because the precursor and trailing films have the same thickness, so there is no net loss or gain of plug volume and the plug length, *L*_p_, does not change. The film thickness is a weak function of *L*_p_ and Re, but depends strongly on Ca (actually, the film thickness is computed for a semi-infinite plug, a good approximation when the plug length *L*_p_ is larger than the tube diameter). This lossless propagation implies that in our model, a plug passing through airways that are already coated with a precursor film (from previous plugs) would not lose any volume (Fig 6C). Only when this plug reaches airways that were never coated previously, does it start losing a fraction of its volume (Fig 6D). Our numerical model accounts for that specific feature of the multiple aliquot delivery.

### Assessing the performance of surfactant delivery

Following [31], we define two dimensionless indices to assess the performance of the delivery, namely the *efficiency index* and the *homogeneity index*. The efficiency index *η* is defined as the percentage of the initially instilled dose volume *V*_*D*_ that actually reaches the ends of the terminal airways:

$$\eta =100\times \frac{\sum_{i=1}^{M}V_{i}}{V_{D}},$$ (6)

*M* being the number of terminal airways and *V*_*i*_ being the volume of surfactant mixture reachingterminal airway #*i*. As for the homogeneity index *HI*, it is computed as the reciprocal of the standard deviation of the normalized distribution of *V*_*i*_:

HI=(〈VN,i2〉−〈VN,i〉2)−12=(M∑i=1MVi2(∑i=1MVi)2−1)−12whereVN,i=Vi1M∑j=1MVj (7)

A vanishing standard deviation, hence an infinite value of *HI*, means that the delivery is perfectly homogeneous. A value of *HI* smaller than 1 corresponds to a poorly homogeneous distribution. The smallest homogeneity is achieved when the entire delivered volume goes to one terminal airway only, in which case $HI=1/\sqrt{M-1}$.

### Instillation conditions and surfactant properties

Simulations were performed for various instillation conditions, in order to compare them with two different sets of experiments of surfactant delivery in rats: first, a set of experiments carried out by Cassidy *et al*. in 2001 on Wistar rats [43]. For this comparison, we tried to match as closely as possible the dimensionless numbers (Re~160, Ca~0.22, Bo~0.6) characterizing the delivery. The second set of experiments was performed by our team and are presented in this paper. In both types of simulations, the tidal volumes are 8 mL.kg^-1^ and 6 mL.kg^-1^, respectively, which are moderate [44]. Such tidal volumes with their frequencies and inspiratory to expiratory time (I:E) ratios correspond in our simulations to flow rates of 26.8 and 6 mL.kg^-1^.s^-1^, respectively.

We simulated surfactant delivery in left lateral decubitus posture (LLD) and right lateral decubitus (RLD). In LLD, the rat is lying on left side and the trachea is horizontal. RLD is similar but on the right side. When these postures are used together (noted L+R), the total dose is divided in 2 half-doses, one half-dose being delivered in RLD posture, and the second half in LLD posture.

Simulations were run for a wide range of dose volumes per kilogram, from 1 to 8 mL.kg^-1^ [18,45–48]. The recommended instilled dose volume per kg of body weight depends on the type of surfactant and on its phospholipid concentration [13] (see Table 2). Surfactant viscosity (Survanta, Infasurf, and Curosurf) is taken equal to *μ* = 30 cP [49,50], density is water-like, i.e., *ρ* = 1 g/mL, and the surface tension is *σ* = 30 dyn.cm^-1^ [51,52].

**Table 2:** Surfactant properties [49,50].

Surfactant	Surfaxin	Exosurf	Survanta	Infasurf	Curosurf	Alveofact
**Phospholipid concentration (mg.mL**^**-1**^**)**	30	13.5	25	35	80	40
**Dose volume/kg** **(mL.kg**^**-1**^**)**	5.8	5	4	3	1.25 or 2.5	1.2
**Molecular dose** **(mg.kg**^**-1**^**)**	175	67.5	100	105	100 or 200	50

### Experiments

All experiments were conducted with approval from the State University of New York Upstate Medical University Institutional Animal Care and Use Committee.

The effect of increasing volumes of surfactant on lung distribution was studied in the rat lung. Infasurf surfactant (210 mg) was tagged with Green Tissue Marking Dye (Green Dye: WAK-Chemie Medical GmbH, Germany) at a concentration of 1% of the total volume of surfactant [53]. Three doses of surfactant (1.125, 2.5, and 5.8 mL.kg^-1^) were tested. Dye and surfactant mixture were incubated at 37°C until the time of distribution. Male Sprague-Dawley rats (n = 3) weighing ~450 g were anesthetized with 1 mL.kg^-1^ of Ketamine/Xylazine I.P. and surgically prepared with a tracheotomy. Rats were then mechanically ventilated with a 6 mL.kg^-1^ tidal volume (V_t_), 2cmH_2_O positive end-expiratory pressure (PEEP), 21% inspiratory oxygen fractionation (FiO_2_), and a frequency of 20 breaths per minute (Dräger Medical, Evita Infinity V500) [54]. The I:E ratio was 1:2 so the flow rate was 6 mL.kg^-1^.s^-1^. The abdominal aorta was then cut to exsanguinate the animal.

Briefly, the animal was disconnected from the ventilator and positioned in left lateral decubitus and reverse Trendelenburg. One half of the surfactant/dye mixture was distributed by sliding a catheter into the endotracheal tube and forming a plug [43]. After 20 mechanical breaths the animal was put in right lateral decubitus and reverse Trendelenburg and the procedure repeated. The lung was then clamped at inspiration and the lungs were excised and immersed in 10% formalin for histological examination.

## Results

A direct comparison between numerical simulations and experimental results is very complicated, due to the difficulty of reliably measuring the amount of surfactant left coating each airway, or the volume of surfactant reaching each terminal airway. One is therefore constrained to compare macroscopic quantities or features that can be practically observed or measured experimentally. In the following, we have chosen two sets of experimental data that illuminate two important features of surfactant delivery: (1) the role played by the dose volume V_D_, due to the existence of a coating cost, V_CC_, predicted by our model; (2) the consequence of multiple aliquot delivery on the efficiency of the delivery.

Before testing our model of these two features, we first want to stress the importance of using an accurate geometrical model of the conducting airway system by comparing two models, one symmetric and one asymmetric, of rat lung. It is noteworthy that this is the first time our model is being tested on asymmetric trees.

### The role of geometry: Symmetric vs. asymmetric lung

**Fig 7:**
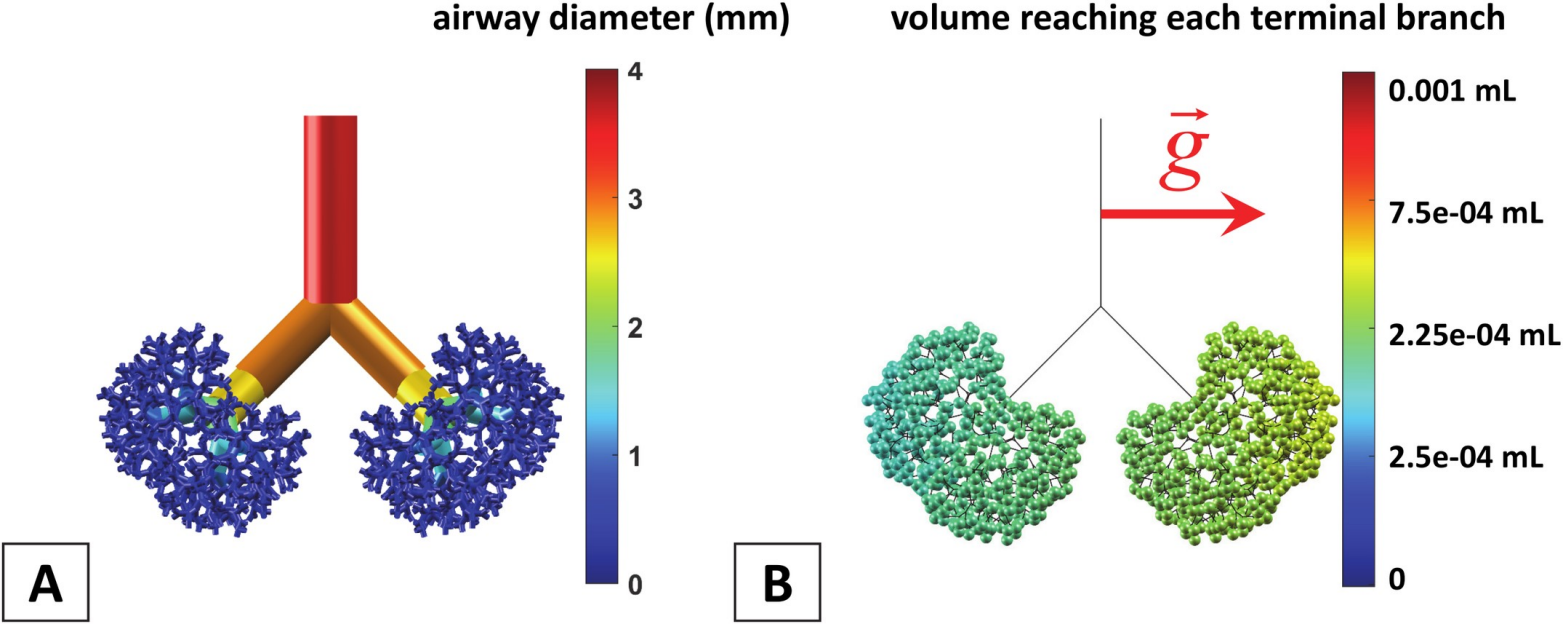
Simulation of surfactant delivery in a symmetric model of the rat airway tree. The simulation is performed on a 0.330-kg rat in LLD position. Surfactant viscosity is μ = 30 cP, dose volume per kg is 1 mL.kg^-1^, and flow rate per kg is 30 mL.kg^-1^.s^-1^. A) Front view of the symmetric model of the rat conducting airways. The color-coding represents the diameter value. B) 3D representation of the delivery (gravity is signaled by the red arrow). The color of each bubble corresponds to the volume of the surfactant reaching each terminal branch. The computed efficiency index is *η* = 26.8% and the homogeneity index of this distribution is *HI* = 7.79.

The asymmetric monopodial structure of the rat lung is one the main factors influencing the inhomogeneity of the end distribution of surfactant. To assess the effect of this asymmetry, we have first compared our asymmetric model to a symmetric version of the rat airway tree. In the latter, all airways pertaining to the same generation share the same diameter and length, which are the average length and diameter at each generation of the corresponding asymmetric airway tree. Each bifurcation is symmetrical, with equal and opposite rotation angles between successive bifurcation planes (+90° and -90°). The branching angle 2θ is 90°. An interesting feature of these two models (symmetric and asymmetric) is that they both have almost the same total surface area. Fig 7A presents the 3D geometry of this symmetric tree. Fig 7B displays a 3D color-coded view of the end distribution of surfactant for a single instillation delivery. In this simulation, the surfactant viscosity is *μ* = 30 cP, the dose volume is 1 mL.kg^-1^, the flow rate is 30 mL.kg^-1^.s^-1^, and the rat is in LLD posture. The computed efficiency index is *η* = 26.8% and the homogeneity index of this distribution is *HI* = 7.79.

**Fig 8:**
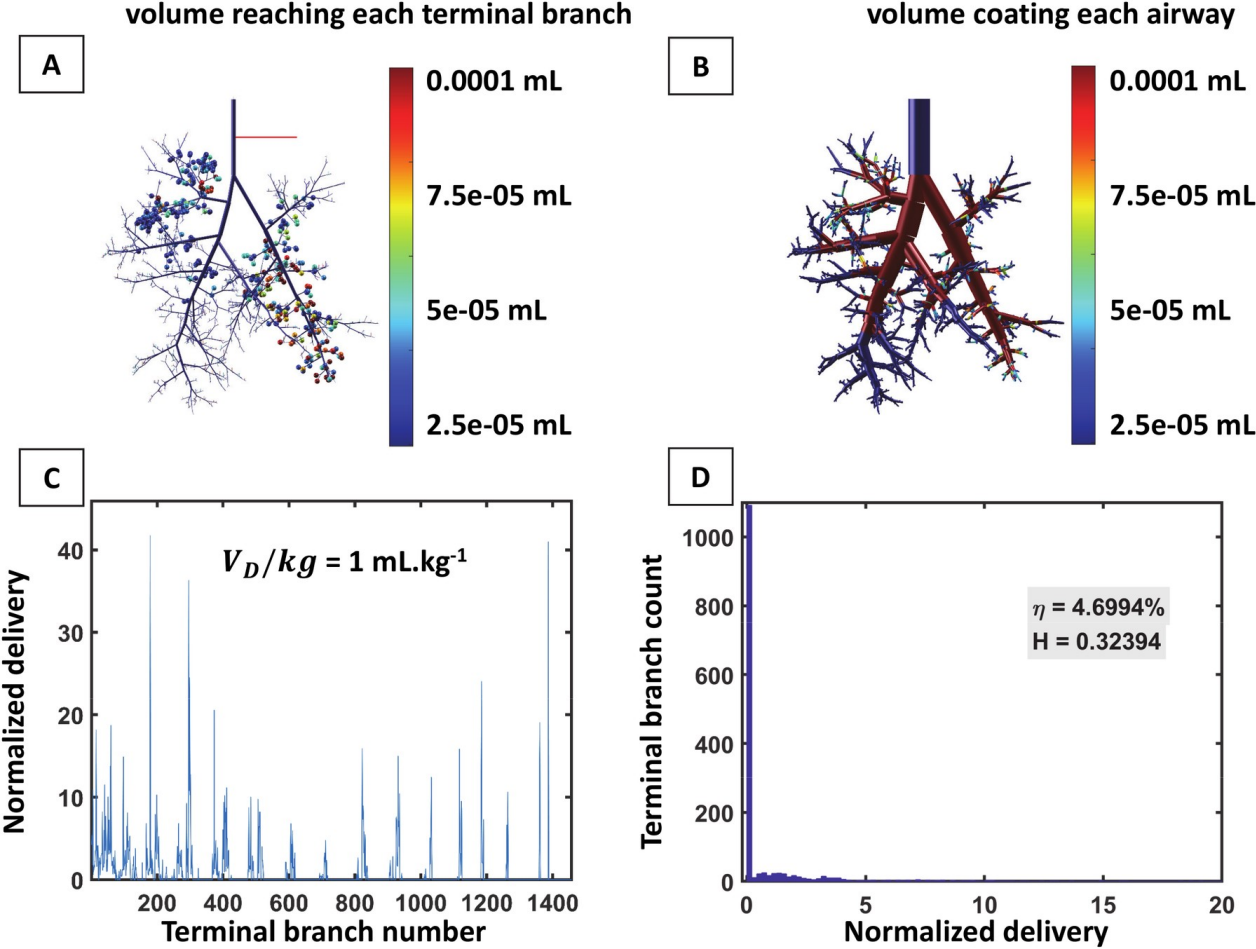
Simulation of surfactant delivery in a realistic model of the rat airway tree. The delivery is performed on a 0.330-kg rat in LLD position. Surfactant viscosity is *μ* = 30 cP, dose volume per kg is 1 mL.kg^-1^, and flow rate per kg is 30 mL.kg^-1^.s^-1^. The airway tree model has 30 generations and 1457 terminal branches. A) 3D representation of the delivery. Each bubble represents a terminal branch and the color of each bubble corresponds to the volume of surfactant reaching the corresponding terminal branch. The computed efficiency index is *η* = 4.69%. B) Coating in the tree. The color-coding represents the volume coating each airway. C) Normalized delivery *V*_*N*_*(i)* plotted vs. the terminal branch number. D) Histogram of normalized delivery. The homogeneity index of this distribution is *HI* = 0.32, which corresponds to a highly inhomogeneous distribution.

We compared this simulation to another simulation of delivery carried out in the same conditions in a realistic asymmetric model of the rat pulmonary airway tree. Fig 8 presents the result of a single instillation at the end of the trachea of a 1 mL.kg^-1^ dose volume per kg in LLD posture. Fig 8A displays the front view of a 3D tree with 1457 terminal airways. At the end of each of these airways, a sphere is color-coded according to the fraction of the initial dose delivered to this specific end (delivered dose divided by the initial instilled dose volume). In this case, the computed efficiency index is about 4.69%. Fig 8B presents a 3D view of the surfactant left coating the airways for the same simulation, the color-coding representing this time the ratio of volume coating each airway to the initial instilled plug volume. The coating cost (the total volume left on the airway walls) is 0.32 mL (95% of the initial instilled volume). Moreover, the end distribution of surfactant is far from being homogenous. In fact, many terminal airways do not receive any surfactant at all as one can see on Fig 8C which displays the end distribution of normalized delivered volumes (for each terminal airway, the volume of surfactant at the end of this airway divided by the sum of all end volumes). The histogram of these normalized volumes, shown in Fig 8D, confirms the highly non-homogeneous distribution, characterized by a very low homogeneity index *HI* = 0.32.

In summary, the efficiency and the homogeneity indices (*η* and *HI*) of the symmetric tree are about 5.7 and 24 times larger than the ones found for the asymmetric tree, respectively. Moreover, all acini of the symmetric tree receive surfactant, which is not at all the case in the asymmetric tree. This comparison underlines the importance of accurately accounting for the asymmetrical monopodial structure of the airway tree when dealing with fluid transport.

**Fig 9:**
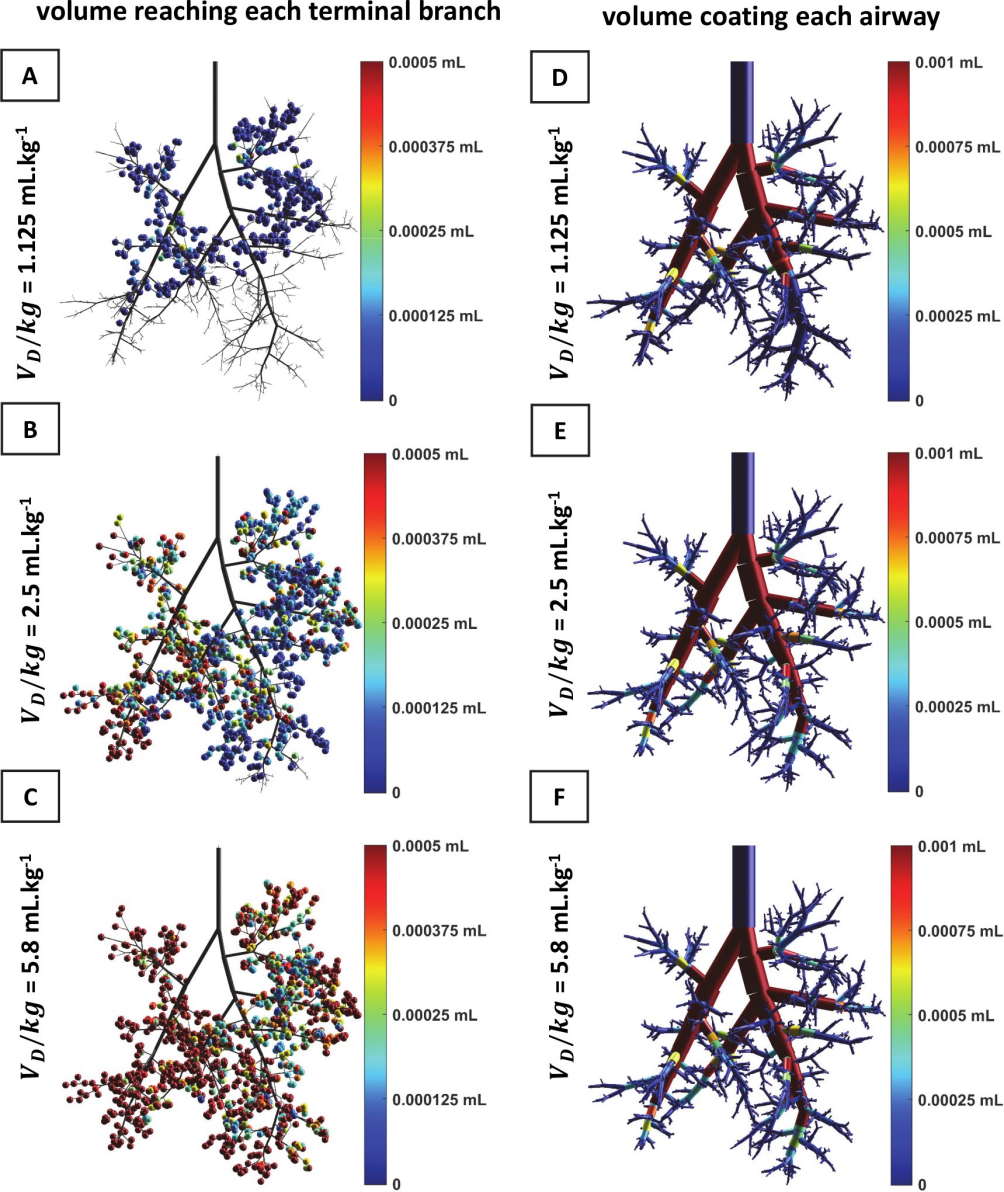
Simulated distributions of surfactant delivery in a 0.330-kg rat in L+R decubitus positions. Surfactant viscosity is μ = 30 cP, and flow rate per kg is 6 mL.kg^-1^.s^-1^. (This corresponds to the experiments presented in **Fig 10**, with 20 breathes per minutes, a tidal volume per kg of 6 mL.kg^-1^, and a ratio between inspiratory and expiratory times I:E = 1:2). The airway tree is asymmetric, with 30 generations and 1457 terminal branches. A) 3D Front view representing the distribution of volumes reaching the terminal branches for a dose volume per kg of 1.125 mL.kg^-1^. The efficiency index is *η* = 5.9%. B) Same figure as (A), for a dose volume per kg of 2.5 mL.kg^-1^. The efficiency index is *η* = 45.6%. C) Same figure as (A), for a dose volume per kg of 5.8 mL.kg^-1^ with efficiency index of *η* = 76%. D) 3D front view displaying the amount of surfactant left coating the airways, in simulation (A). The color-coding represents the volume left coating each airway. The homogeneity index is *HI* = 0.2. E) Same figure as (D), corresponding to simulation (B). The homogeneity index is *HI* = 0.29. F) Same figure as (D), corresponding to simulation (C). The homogeneity index is *HI* = 0.28.

*Simulating the experiments*: Using the asymmetric model of the rat conducting airways developed and tested above, we now compare our simulations to our experiments. First, we set the flow rate to 6 ml.kg^-1^.s^-1^ and then examine the applied dose volumes per kg of V_D_/kg = 1.125, 2.5, and 5.8 mL.kg^-1^ to explore the role of V_D_. Fig 9A, 9B and 9C present 3D views of the end distributions of surfactant in this rat lung after a divided instillation, half L and half R lateral decubitus position. One observes that increasing V_D_ allows more surfactant to reach the terminal regions. Indeed, the efficiency is raised from 5.9% to 45.6% and 76%, respectively (about ten-fold increase!), whereas the homogeneity index remains very poor, about 0.2 to 0.29. The amount of surfactant left coating the airways is displayed for all dose volumes per kg in Fig 9D, 9E and 9F. In the last two cases, the coating cost V_CC_ is about 0.45 mL, only 30% more than the one found for a 1.125 mL.kg^-1^ instilled dose volume per kg, whereas the amount of instilled dose volume per kg is 2 and 5 times larger, respectively. V_CC_ reaches a plateau above a given initial dose volume per kg.

**Fig 10:**
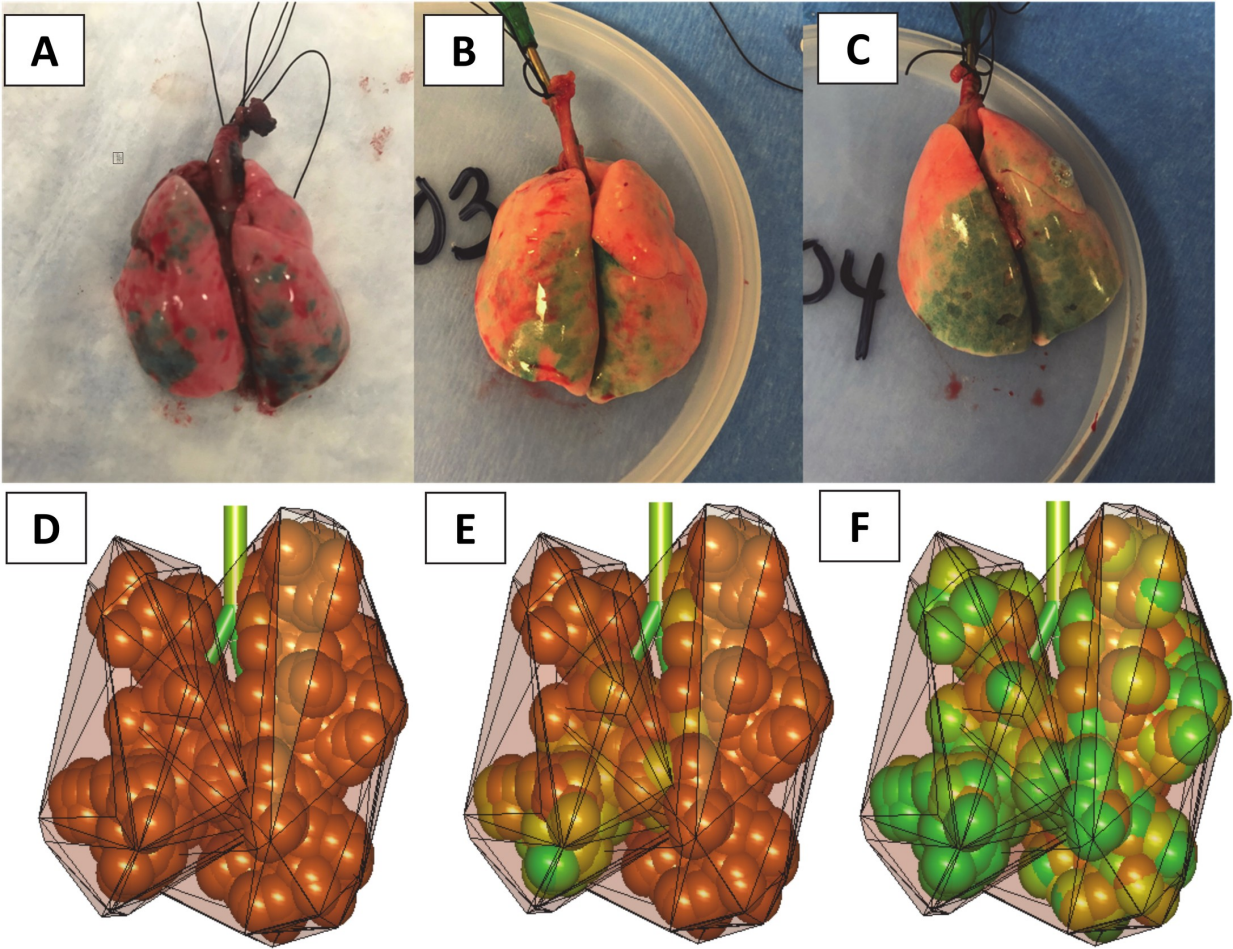
Delivery of three surfactant dose volumes into a rat lung (L+R posture). Surfactant (Infasurf) was tagged with Green Tissue Marking Dye. A) Dose volume per kg = 1.125 mL.kg^-1^, B) Dose volume per kg = 2.5 mL.kg^-1^, and C) Dose volume per kg = 5.8 mL.kg^-1^. D) to F) Simulation results in the same conditions: a sphere is located at the end of each terminal branch, of the size of an acinus. The color of the sphere ranges continuously from fully orange (if no surfactant has reached the terminal branch) to fully green (if the amount of surfactant is larger than 10^−3^ mL).

When looking now at the experiments, one can see a marked visual difference in surfactant distribution between the three dose volumes per kg (Fig 10A–10C). The low dose volume per kg (1.125 mL.kg^-1^) showed scattered heterogeneous distribution in both right and left lungs. Increasing V_D_/kg to 2.5 mL.kg^-1^ resulted in a higher concentration of surfactant in the caudal portions of both lungs. For the highest V_D_/kg (5.8 mL.kg^-1^) an even distribution of surfactant in the caudal portion of both lungs was observed, while the remaining lung showed no surface distribution at all. In summary, as in the simulations, the low dose volume per kg resulted in small areas of heterogeneous surfactant distribution whereas the high dose volume per kg induced a more locally homogeneous distribution isolated to the caudal portion of both lungs, indicating regional-scale heterogeneity. Of course, the heterogeneity observed here lies at the outer surface of the pulmonary airway system which corresponds to the most distal part of the external acini. It is reasonable to assume that having surfactant reaching this surface implies that the corresponding acini are also filled with surfactant, and that this heterogeneity reflects the 3D patchy distribution of surfactant in the lung volume. Our simulations (Fig 10D–10F) support this assumption.

**Fig 11:**
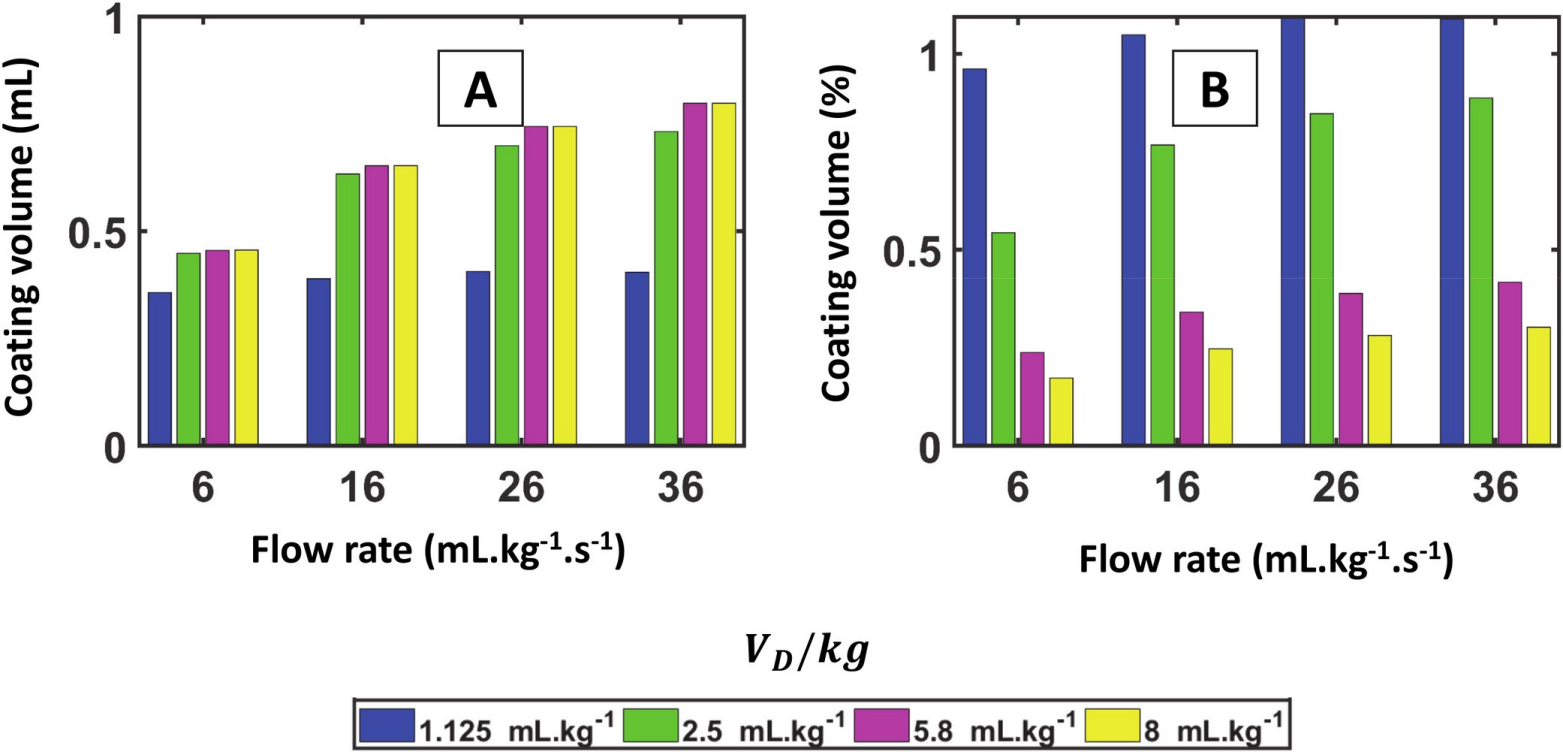
Total coating cost vs. flow rate for a 0.330-kg rat lung in L+R position. A) Absolute amount of coating volume in the entire tree. The coating volume (or coating cost) reaches a plateau for larger dose volume per kg and at higher flow rate. B) Coating volume divided by the initial instilled dose volume. The coating volume represents a smaller fraction of the initial volume at larger dose volume per kg.

Changing the flow rate in the simulations does not significantly alter the coating cost: one can see from Eq 2 that the coating thickness reaches a limit value at high velocity, namely about 0.36 times the airways radius. This saturation of the coating cost is shown in Fig 11A (absolute amount) and in Fig 11B (fraction of the initial volume). Fig 11A illustrates that, for a given flow rate, the total coating volume is almost identical for different dose volumes. Fig 11B shows that the ratio V_CC_/V_D_ slowly increases with the flow rate. This is due to the thicker trailing film left by the plugs propagating through the tree. At the same time, due to the increased velocity, the influence of gravity decreases and the splitting factor gets closer to 0.5. However, if V_D_ is too small, only a small number of terminal airways are effectively supplied with surfactant which means that both efficiency and homogeneity plummet, independently of the velocity.

### Multiple instillations: Comparison with experimental results

**Fig 12:**
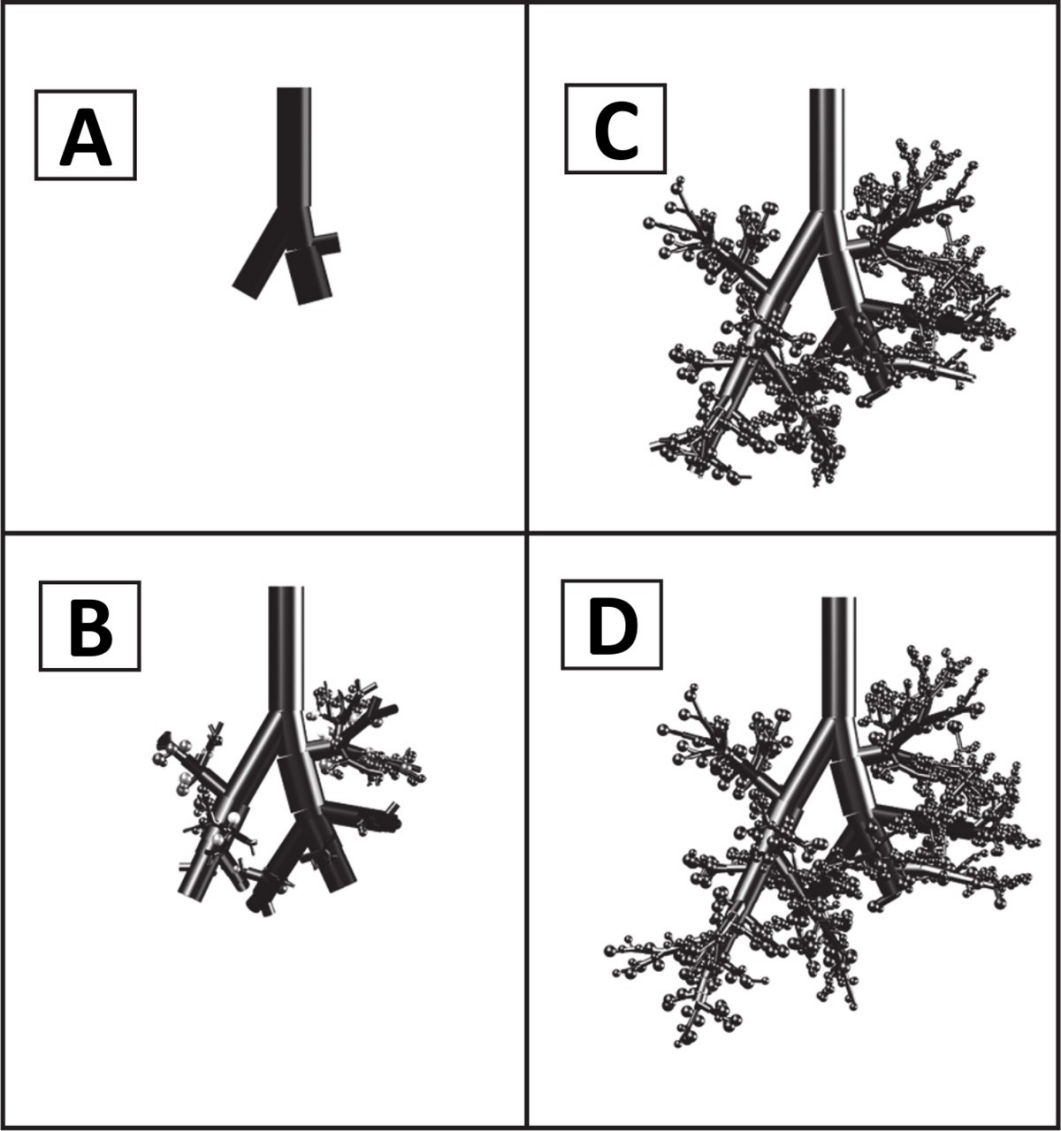
**Our surfactant delivery simulation (A-D) at the end-inspiration** 1^st^ (A), 3^rd^ (B), 6^th^ (C), and 10^th^ (D) aliquot. These images have to be compared with the corresponding experimental observations (images E-F) of ref. **[43]**.

**Fig 13:**
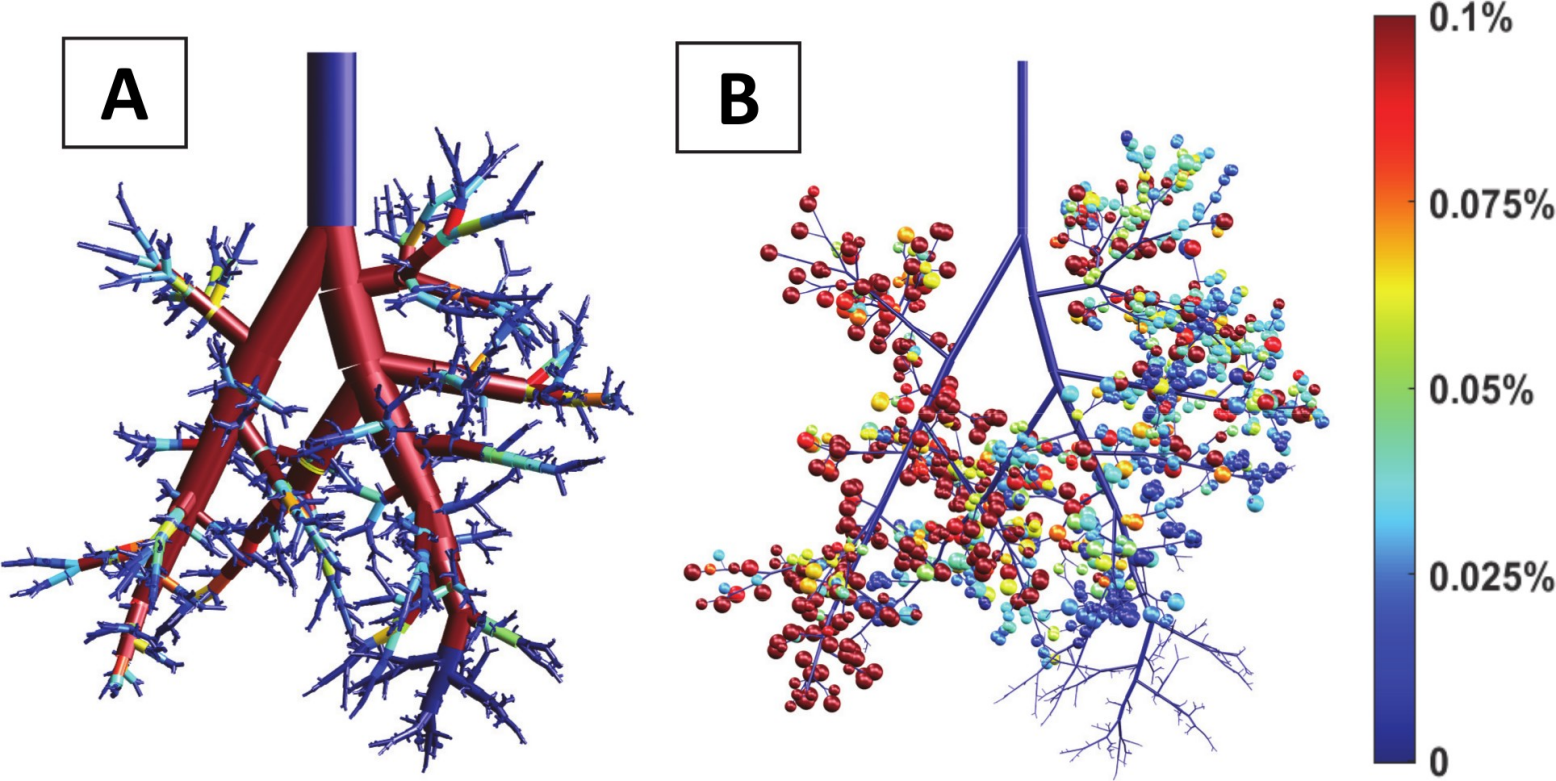
Simulations of multiple aliquot surfactant delivery. A) Front view of the simulated end distribution of surfactant in a 3D model of rat lung (10^th^ aliquot), to be compared with **Fig 8H** of ref. **[43]**. The color-coding represents the volume coating each airway divided by the initial instilled dose volume. B) Delivered surfactant to the terminal airways. The color of each bubble corresponds to the percentage of the initial dose volume reaching each terminal airway.

A very interesting option for improving the efficiency surfactant delivery in the more distal regions is the multiple aliquot approach. Here we compare numerical simulations run on our numerical model to experimental results obtained by Cassidy *et al*. on Wistar rats [43]. Simulations of surfactant delivery have been carried out using the same viscosity, surface tension, volume, posture, and velocity than in the experiments (see Table 3). Surfactant is being delivered in multiple aliquots, 0.053 mL per breath (0.1 mL.kg^-1^) during the first 10 breaths (which amounts to 1 mL.kg^-1^ in total). At the end of each inspiration, images of the lung were captured. Fig 12 shows our simulations of the end distributions of surfactant mixture (A-D) after the 1^st^, 3^rd^, 6^th^, and 10^th^ breath, to be compared to the distributions observed experimentally in ref. [43]. In both cases, one can observe that the liquid plugs propagate increasingly deeper down the airway tree during each successive breath. This is particularly striking in Fig 13 which displays the surfactant delivered during the 10^th^ breath, to compare with Fig 8H of ref. [43]. The efficiency of the delivery is about 59%, which is quite good. This high efficiency is due to the pre-existing film lining the airways which allows the plugs to propagate without losing most of their mass.

**Table 3:** Properties and condition of instilled surfactant in simulations and experiments [43].

	Simulation (Long-Evans)	Experiment (Wistar)
**Viscosity (cP)**	12.2	12.2
**Surface tension (dyn.cm**^**-1**^**)**	54	54
**Density (g.cm**^**-3**^**)**	1.22	1.22
**Flow rate/kg (mL.s**^**-1**^**.kg**^**-1**^**)**	26.8	16
**Dose volume/kg (mL.kg**^**-1**^**)**	1 per breath (10 breaths)	1 per breath (10 breaths)
**Position**	vertical	vertical
**CapillaryNumber (trachea)**	0.22	0.22
**BondNumber (trachea)**	0.64	0.58
**ReynoldsNumber (trachea)**	165	160
**Trachea diameter (cm)**	0.34	0.324
**Velocity (cm.s**^**-1**^**) (trachea)**	97	97
**Rat weight (kg)**	0.330	0.500

**Fig 14:**
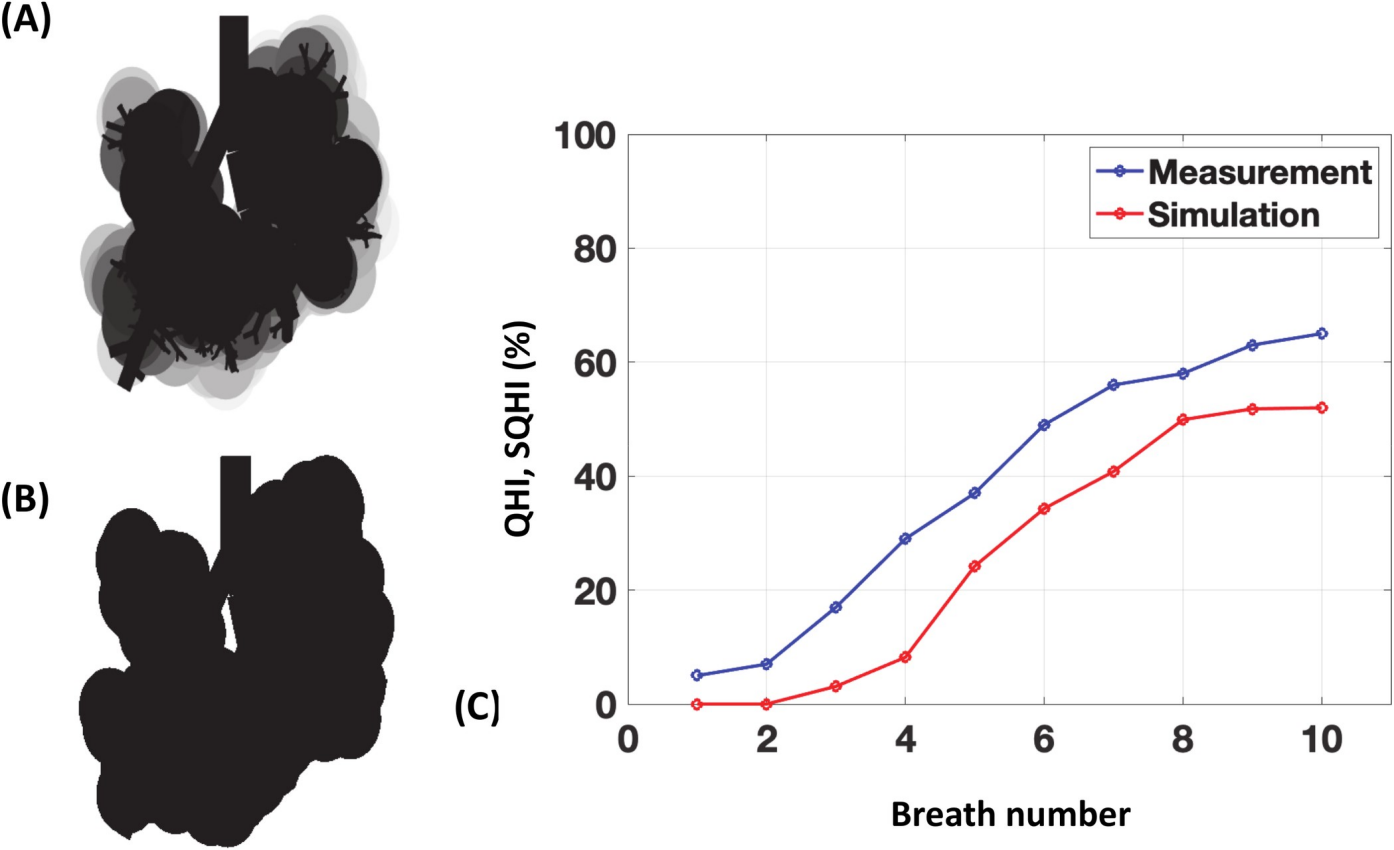
Comparison between measured Quadrant Homogeneity Index (QHI) [43] and simulated Quadrant Homogeneity Index (SQHI). A) Image computed from the numerical simulation to mimic the images of **Fig 12**. A sphere is plotted around each terminal branch. Its grayscale goes from white (no surfactant) to black (fully coated). When two spheres superimpose, their grey levels are added. B) The image obtained in frame (A) is thresholded to obtain a black and white image (threshold is 90% grey), on which the SQHI can be computed as in **[43]**. C) Comparison of measured QHI and simulated QHI on 10 consecutive breaths.

In the experiments carried out by Cassidy *et al*. [43], the homogeneity of the end distribution of surfactant was initially quantitatively assessed using a different method: the lung would be divided into four main quadrants; in each quadrant the two-dimensional area reached by the liquid would be measured. Each of these areas would be then divided by the total surface area of the quadrant (*AR*), and the homogeneity index defined for each image as the smallest value of *AR* divided by its largest one. In what follows, we will call this quantity comprised between 0 and 1 the “quadrant homogeneity index” (*QHI*). A *QHI* of 1 means a perfectly homogeneous distribution (at the size of a quadrant) while a vanishing *QHI* means a strongly inhomogeneous distribution. This *QHI* index differs from the homogeneity index (*HI*) introduced later in [31] and defined by Eq (2), which is more general and captures more details about the distribution homogeneity. To allow a direct comparison between experiments and our model, we have computed a tentative estimate of the *QHI* from our simulations. To that end, simulated images of the lung are created by placing a sphere around each terminal branch of the bronchial tree (see Fig 14). The size of the sphere is constant and corresponds to the average size of an acinus. The sphere is colored on a gray scale, a white color meaning that no surfactant has reached the acinus and while black means that the amount of the surfactant reaching the acinus is sufficient to coat the entire acinar surface. Table 4 displays the efficiency and the homogeneity indices computed from our simulations, the *QHI* obtained measured in the experiments, and the *SQHI* computed from our simulations. We see that the efficiency rises at each breath, from 0% to 59% in the 10^th^ breath. The homogeneity rises also, whether measured by our *HI* index or by the original quadrant-based index. We can observe that *QHI* and *SQHI* follow very similar curves, with a slight shift that is probably due to a difference in the initial tracheal coating, difficult to estimate from the original images.

**Table 4:** Simulated efficiency and homogeneity indices compared with the homogeneity index measured in Cassidy *et al*. [43].

Breath number	1	2	3	4	5	6	7	8	9	10
**Efficiency (%)**	0	0	2	10	20	31	41	48	54	59
**Homogeneity Index (*HI*)**	0	0	0.19	0.39	0.53	0.65	0.72	0.76	0.78	0.8
**Quadrant Homogeneity Index (*QHI*) (%) **[43]	5	7	17	29	37	49	56	58	63	65
**Simulated Quadrant Homogeneity Index (*SQHI*) (%)**	0	0	3.1	8.2	24.2	34.3	40.8	49.9	51.8	52

**Fig 15:**
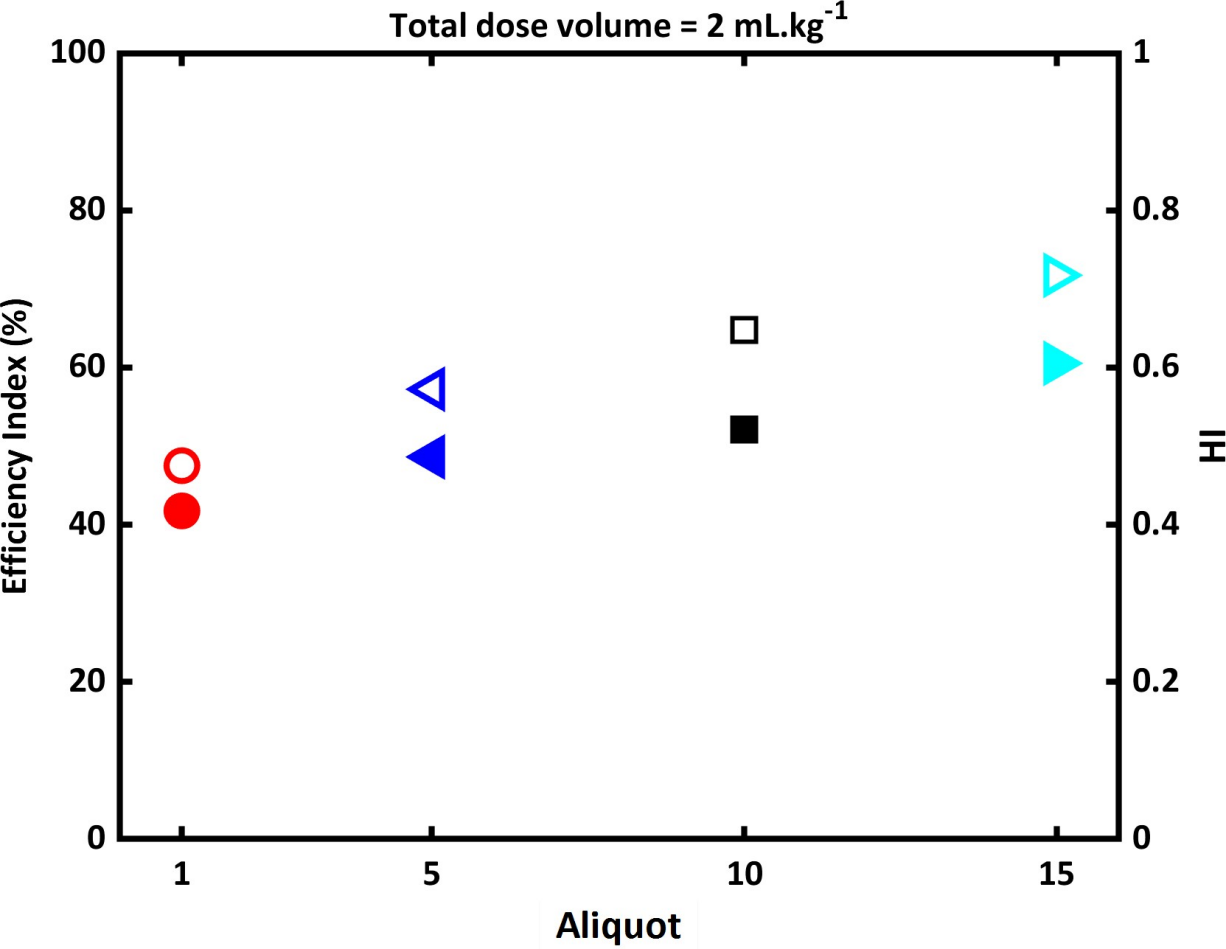
Efficiency index *η* (open symbols) and homogeneity index *HI* (filled symbols) in different aliquot initial dose volume, computed in a 0.330-kg rat lung (total dose volume: 2 mL.kg^-1^, flow rate: 30 mL.kg^-1^.s^-1^, LLD, viscosity *μ* = 30 cP).

Finally, we study in the simulations the result of an increased initial dose volume per kg, from 1 to 2 mL.kg^-1^. Fig 15 displays the efficiency and homogeneity indices computed for 1, 5, 10, and 15 aliquots, respectively. We observe a clear increase of both indices for a larger number of aliquots. However, the homogeneity index remains always smaller than 1 which indicates very poorly homogeneous distributions. This poor homogeneity is confirmed visually in the experiments, at all dose volumes. The origin of this inhomogeneity relatively insensitive to the dose volume is to be found in the monopodial airway structure (i.e., the strong branching asymmetry): increasing the dose volume simply increases the delivery to the favored alveoli.

## Discussion

This study is the first attempt to validate with animal data the numerical and mathematical model initially introduced in [31–33] and used to simulate surfactant delivery in human lung models. To this end we have developed realistic 3D models of the rat tracheobronchial tree, accounting in particular for the branching asymmetry, which is crucial for a reliable computation of liquid plug propagation.

Our initial numerical study of SRT in human [31] had shown that, contrary to the adult lung, the neonate lung is a well-mixed compartment for surfactant delivery. Due to the respective scaling of gravity force vs. surface tension and to the much larger surface of airway walls in the adult human lung than in the neonate (more than 3000 cm^2^ for the adult and about 1500 cm^2^ for the 3-month old neonate [55]), the coating cost becomes vastly larger than delivered dose in the adult.

The simulations of surfactant delivery in rat lungs presented here offer a different and more complex picture. Despite the small size of the rat lung (smaller than the human neonate), simulations of delivery under realistic conditions result in a poor efficiency index (e.g. 4.69%) and a very small homogeneity index (0.32), overall a very poor performance. This seems to contradict our preceding explanation based on the size. The reason for this discrepancy can be found in the asymmetric structure of the airway pulmonary tree of the rat. Our computations emphasize that, beyond its size, the detailed geometrical structure of the lung airways plays a crucial role in distributing fluid to the alveolar region. The splitting factor, α, at a bifurcation depends on asymmetries due to orientation with gravity and also geometry. For the rat lung, it is this geometric asymmetry that can dominate. It is interesting on this topic to point out that the descriptions of the rat pulmonary airway tree found in the literature are generally not suited for the simulation of liquid transport. These descriptions are usually used for simulating

1. Air and gas transport, for which one essentially needs only the airway diameters and lengths. For this entire airway tree, it is even more efficient to store the diameter and length of the trachea, then the ratios between successive generations of these parameters. Angles are almost of no interest as they have little influence on the gas transport.
2. Aerosol deposition, for which not only the airway diameters and lengths are needed, but also the branching angles at each bifurcation. Deposition by impaction occurs essentially at the bifurcation carina, which explains the role played by the branching angle. On the contrary, rotation angles between successive bifurcation planes play very little role [56]. Angles to gravity need only to be stored statistically, as they are involved in sedimentation.

Simulating liquid delivery in the tracheobronchial tree, coupled with the air is a two-phase flow, which is far more demanding in terms of knowledge of the geometry. One needs now of course airway diameters and lengths, but also precise values of the angles to gravity for each airway and each bifurcation. These parameters are not usually found in the literature or in the commonly available lung airway models, hence the need to develop an original 3D model of the rat pulmonary airway tree. Our simulations show that the delivery reflects the cranio-caudal asymmetry of the rat lung, a conclusion qualitatively consistent with the experimental data.

Our model also suggests that one could customize the delivery for each patient by accounting precisely for the branching asymmetry in the first few generations, either to improve the final homogeneity or to reach one lobar or sub-lobar region. It therefore opens the way of engineering the delivery to target specific regions of the lung by tuning the initial dose volume, the flow rate, and the patient posture.

In addition to the role played by asymmetry in the pulmonary airway systems, our simulations confirmed that the instilled dose volume is a critical parameter. This observation was corroborated with the biologic data of surfactant distribution in rat lungs. For dose volumes per kg ranging from 1 to 8 mL.kg^-1^, the coating cost (the amount of surfactant left lining the airways walls) is very stable, about 0.32–0.76 mL in the rat asymmetric lung (L+R posture). When increasing even more the initial dose volume, the coating cost reaches a plateau. Once this plateau is reached, the efficiency of the delivery improves for larger dose volumes. This phenomenon seems to be universal and consistent with what had been already observed in our first computation in the human lung. However, a way to overcome this coating cost is not necessarily to increase the dose volume.

Indeed, a new feature of the model presented here is the possibility to simulate for the first time multiple-aliquot delivery. A plug travelling over a previously coated airway (by a previous plug) will not lose its mass until it reaches regions of the lung that were not attained by the preceding plugs. Our simulations clearly show that resorting to multiple aliquot delivery without changing the posture increases the efficiency. Increasing the homogeneity would require changing posture, but then the benefit of the multiple aliquot delivery is partially lost, the subsequent plugs entering parts of the lung which have never been visited during the preceding instillations.

Based on this collection of results, one could wonder what are the good strategies for a successful delivery. We have seen the major role played by the dose volume. It is therefore natural to use this tuning parameter to target specifically some regions of the lung. If the region to be treated is the conducting airway, then a small V_D_ and a high flow rate would optimally distribute the surfactant everywhere in the lung while maximizing the deposition on the airway walls. If the target on the contrary is located distally within a well-defined lobe, one will favor a larger V_D_ and a small flow rate, able to propagate the liquid plugs for a long time without losing too much of their masses along the way. The dependence of homogeneity and efficiency index vs. the dose volume remains however similar to the symmetric case.

We can envision further improvements of our model along several directions. While we have studied the detailed effects of surfactant on liquid plug propagation, trailing film thickness, and rupture in a single tube or channel [38,42,57–60], accounting for surface-active effects, fluid inertia in the deposition process would be a next step when dealing with a branching tree geometry. The general effect of lowering the constant surface tension is used instead. For example, rather than 70 dyn/cm we use 30 dyn/cm. In addition, we have explored 2D plug splitting and its effects on the split ratio [61]. The fate of surfactant once it coats the airways or has entered the acinus is also a challenging field of study. Understanding how surfactant will progressively coat the entire complex acinar surface requires accounting for the dynamics of alveolar recruitment during the breathing cycle [62], the mechanics of the compliant distal airways [63], and the complex motion of surfactant plugs in the rough-walled acinar ducts [64]. Accurately modeling this step should provide important insights into the delivery process, the final distribution of surfactant on the alveolar walls, and the global efficiency of SRT.

## Conclusion

In summary, we have presented a numerical model of surfactant plug propagation into the rat pulmonary airway system. To that end, we have first developed an original 3D asymmetric geometrical model of the rat conducting airways, based on morphometric measurements, dedicated for fluid transport. Using this model, we have assessed the effects of dose volume, flow rate, and multiple aliquot delivery, and observed the existence of a threshold dose volume under which the coating cost represents 100% of the initial dose volume, which means that no surfactant reaches the acinar region at all. Moving from single to multiple instillations for a given total dose volume allows to overcome partially the constraint of the coating cost and to globally improve the efficiency.

Our results also underline the crucial role played by the detailed 3D geometrical structure: it appears as one of the important determinants of the end distribution of surfactant (especially when moving from a symmetric to an asymmetric model). In particular, despite the small size of the animals, the end distributions of surfactant in rat lungs are very poor, both in our simulations and in the experiments. This high inhomogeneity is mainly due to the asymmetric architecture of the rat airway tree, very different from that of the human. A cautionary note is that pig and sheep lungs also have monopodial architecture and have been used for SRT experimentation [65,66]. Thus, generalizing results of surfactant delivery between different species might be misleading, even for mammals of similar size or weight. The specific geometry of the studied species has to be accounted for, in order to under the precise quantitative results obtained from numerical models. In addition, material properties of surfactant such as viscosity and surface tension may drastically alter the efficiency. This dependency makes it more complicated to compare results of trials performed with different surfactant. Preliminary data on surfactant dose volume distribution in rats confirm our computational findings.

## Supporting information

S1 Appendix Mathematical model of deposition and plug splitting in a single asymmetric bifurcation. (PDF)
