# Supplementary material for: Surfactant delivery in rat lungs: Comparing 3D geometrical simulation model with experimental instillation
Source: PLoS Comput Biol. 2019 Oct 17;15(10):e1007408. doi: 10.1371/journal.pcbi.1007408 (PMC6818804; doi:10.1371/journal.pcbi.1007408)
Supplement: S1 Appendix — (PDF) [file pcbi.1007408.s001.pdf]

Mathematical model of deposition and plug splitting in a single asymmetric bifurcation

1 Definitions

- a_1, a_2, a_3: diameters of the parent airway and of the two daughter airways, respectively
- λ_2, λ_3: ratios between daughter airway and parent airway diameters ($\lambda_2 = a_2/a_1$, idem for 3)
- θ_2, θ_3: branching angles between parent and daughter airways (can be negative).
- γ: tilt angle, angle between the parent airway and the horizontal plane
- φ: roll angle, angle between a line perpendicular to the parent airway in the bifurcation plane and the horizontal plane
- σ: surface tension between air and surfactant
- μ, ρ: surfactant viscosity and surfactant density, respectively
- P_1: upstream pressure in the parent airway, before the plug
- P_2, P_3: downstream pressures in the daughter airways, after the plugs
- π_1: pressure inside the plug, right at the rear meniscus
- π_2, π_3: pressures inside the plug, right next to the front meniscus in the daughter airways
- V_0: volume of the initial plug entering the parent airway
- V_c: volume (from the initial plug) left coating the parent airway
- V_1: volume (from the initial plug) being splitted between the two daughter airways
- V_2, V_3: volumes of the plugs in the daughter airways right after splitting is complete
- L_1: length of the plug in the parent airway right before splitting

- L_2, L_3: lengths of the plugs in the daughter airways right after splitting
- h: thickness of the coating layer of surfactant in the parent airway
- Q_1, Q_2, Q_3: plug flow rates in the parent airway and in the daughter airways, respectively

2 Step 1: plug propagation in the parent airway

The volume V_1 is the volume of liquid reaching the bifurcation. It is different from the initial volume V_0 which entered the parent airway. The difference is called the *coating volume* V_c. It is the volume left lining the parent airway wall during the progression of the plug:

$$V_1 = V_0 - V_c \tag{1}$$

The thickness h of the trailing film left behind the plug depends on the average velocity of the fluid at the rear meniscus, called U_p (see Zheng 2005), and more precisely on the capillary number Ca_P defined from U_P:

$$Ca_p = \frac{\mu U_p}{\sigma} \quad , \quad \frac{h}{a_1} = 0.36 \left(1 - \mathrm{e}^{-2Ca_p^{0.523}}\right) \tag{2}$$

U_p is related to the velocity U_1 of front meniscus of the liquid plug (at the moment it reaches the bifurcation) through conservation of the flow rate

$$Q_1 = \pi a_1^2 \, U_1 = \pi \left(a_1 - h\right)^2 U_p \tag{3}$$

This means that the volume V_1 that will be finally delivered to the daughter airways is deduced from V_0 (the volume of the plug entering initially the parent airway) through:

$$V_1 = V_0 - \pi \left[a_1^2 - (a_1 - h)^2\right] L_a = V_0 - V_a \left[1 - \left(0.64 + 0.36 \; \mathrm{e}^{-2Ca_p^{0.523}}\right)^2\right] , \tag{4}$$

L_a and V_a being the length and the volume of the parent airway, respectively.

In summary, when a plug of volume V_0 enters the parent airway with velocity U_1 of the front meniscus, one first computes the thickness of the trailing film, and thus the maximal volume V_c of surfactant that will be left lining the parent airway if the plug reaches the end of the airway and goes into the daughter airways. If this coated volume is smaller than the volume of the plug, then $V_1 = V_0 - V_c$ is the volume of surfactant available for splitting.

3 Step 2: splitting at the bifurcation

The splitting ratio is obtained by stating that the downstream pressures in both daughter airways are equal. The pressure difference between the upstream pressure P_1 in the parent branch and the downstream pressure P_2 in the daughter branch 2 is the sum of several contributions:

$$P_1 - P_2 = (P_1 - \pi_1) + (\pi_1 - \pi_0) + (\pi_0 - \pi_2) + (\pi_2 - P_2) \tag{5}$$

- Pressure drop across the rear meniscus

$$P_1 - \pi_1 = \frac{2\sigma}{a_1 - h} \tag{6}$$

- Pressure drop in the parent branch: viscous (Poiseuille) drop + hydrostatic pressure drop

$$\pi_1 - \pi_0 = \frac{8\mu L_1}{\pi a_1^4} Q_1 - \rho g \sin\gamma \, L_1 \tag{7}$$

- Pressure drop in the daughter branch: viscous (Poiseuille) drop + hydrostatic pressure + inertia term

$$\begin{aligned}\pi_0 - \pi_2 =& \frac{8\mu L_2}{\pi a_2^4} Q_2 + \rho g \left(\sin\theta_2 \sin\varphi - \cos\theta_2 \sin\gamma\right) L_2 \\ &+ \frac{1}{2}\rho \left(\frac{Q_2}{\pi a_2^2}\right)^2 - \frac{1}{2}\rho \left(\frac{Q_1}{\pi a_1^2}\right)^2\end{aligned} \tag{8}$$

- Pressure drop across the front meniscus in daughter 2

$$\pi_2 - P_2 = -\frac{2\sigma}{a_2} \tag{9}$$

One introduces here the quantity f_2 (resp. f_3) defined as

$$f_2 = \sin\theta_2 \sin\varphi - \cos\theta_2 \sin\gamma \tag{10}$$

The same equations apply to the daughter branch 3. One has to note that the branching angles θ_2 and θ_3 are algebraic, which means they can take negative values (in fact, for the symmetric bifurcation, $\theta_3 = -\theta_2$).

We now examine the situation when the plug has just entirely passed the bifurcation, the rear meniscus being at the location of π_0. This means that $L_1 = 0$ and that the plug is entirely split in the daughter branches, with lengths L_2 and L_3, respectively. Since both new plugs have passed the bifurcation in the same duration, we can infer that:

$$\frac{L_2}{U_2} = \frac{L_3}{U_3} \qquad \text{hence} \qquad \frac{L_2}{\frac{Q_2}{\pi a_2^2}} = \frac{L_3}{\frac{Q_3}{\pi a_3^2}} \tag{11}$$

One defines the splitting ratio R_s as

$$R_s = \frac{V_2}{V_3} \tag{12}$$

This parameter can range from 0 to $+\infty$, if the entire plug goes to one daughter branch or the other. To handle an always finite and more convenient parameter, one introduces the splitting factor α such that $V_2 = \alpha V_1$ and $V_3 = (1-\alpha)V_1$. It follows immediately that $R_s = \alpha/(1-\alpha)$ and that $\alpha = R_s/(1+R_s)$. From Eq. 11, one deduces the ratio between the flow rates Q_2 and Q_3:

$$\frac{Q_2}{Q_3} = \frac{\pi a_2^2 \, L_2}{\pi a_3^2 \, L_3} = \frac{V_2}{V_3} = R_s \tag{13}$$

Finally, mass conservation tells us that $V_2 + V_3 = V_1$ and $Q_2 + Q_3 = Q_1$.

3.1 Equation on R

One obtains the value of the splitting ratio R by stating that both downstream pressures are equal, $P_2 = P_3$, or that both pressure drops are equal, $\pi_0 - P_2 = \pi_0 - P_3$. It follows that

$$\frac{8\mu}{\pi a_2^4} \, L_2 Q_2 + \rho g f_2 L_2 + \frac{1}{2}\rho \left(\frac{Q_2}{\pi a_2^2}\right)^2 - \frac{2\sigma}{a_2} \tag{14}$$

$$= \frac{8\mu}{\pi a_3^4} \, L_3 Q_3 + \rho g f_3 L_3 + \frac{1}{2}\rho \left(\frac{Q_3}{\pi a_3^2}\right)^2 - \frac{2\sigma}{a_3} \tag{15}$$

Rewriting this equation in terms of volumes leads to

$$\frac{8\mu}{\pi^2 a_2^6} \, V_2 Q_2 + \rho g f_2 \, \frac{V_2}{\pi a_2^2} + \frac{1}{2}\rho \left(\frac{Q_2}{\pi a_2^2}\right)^2 - \frac{2\sigma}{a_2}$$

$$= \frac{8\mu}{\pi^2 a_3^6} \, V_3 Q_3 + \rho g f_3 \, \frac{V_3}{\pi a_3^2} + \frac{1}{2}\rho \left(\frac{Q_3}{\pi a_3^2}\right)^2 - \frac{2\sigma}{a_3} \tag{16}$$

All volumes and flow rates in the daughter branches are now expressed in term of the parent branch:

$$\frac{8\mu}{\pi^2 a_2^6} \left(\frac{R_s}{1+R_s}\right)^2 V_1 Q_1 + \rho g f_2 \, \frac{V_1}{\pi a_2^2}\frac{R_s}{1+R_s} + \frac{\rho}{2}\left(\frac{Q_1}{\pi a_2^2}\right)^2 \left(\frac{R_s}{1+R_s}\right)^2 - \frac{2\sigma}{a_2}$$

$$= \frac{8\mu}{\pi^2 a_3^6} \left(\frac{1}{1+R_s}\right)^2 V_1 Q_1 + \rho g f_3 \, \frac{V_1}{\pi a_3^2}\frac{1}{1+R_s} + \frac{\rho}{2}\left(\frac{Q_1}{\pi a_3^2}\right)^2 \left(\frac{1}{1+R_s}\right)^2 - \frac{2\sigma}{a_3} \tag{17}$$

Multiplying by $(1+R_s)^2$ on both sides yields:

$$\frac{8\mu}{\pi^2 a_2^6}\ R_s^2\ V_1 Q_1 + \rho g f_2\ \frac{V_1}{\pi a_2^2} R_s(1+R_s) + \frac{\rho}{2}\left(\frac{Q_1}{\pi a_2^2}\right)^2 R_s^2 - \frac{2\sigma}{a_2}(1+R_s)^2$$
$$= \frac{8\mu}{\pi^2 a_3^6}\ V_1 Q_1 + \rho g f_3\ \frac{V_1}{\pi a_3^2}(1+R_s) + \frac{\rho}{2}\left(\frac{Q_1}{\pi a_3^2}\right)^2 - \frac{2\sigma}{a_3}(1+R_s)^2 \tag{18}$$

One finally rewrites the equation in R as a second order equation:

$$A\ R_s^2 + B\ R_s + C = 0 \tag{19}$$

$$\text{with} \quad \begin{cases} A &= \dfrac{8\mu}{\pi^2 a_2^6}\ V_1 Q_1 + \rho g f_2\ \dfrac{V_1}{\pi a_2^2} + \dfrac{1}{2}\rho\left(\dfrac{Q_1}{\pi a_2^2}\right)^2 - 2\sigma\left(\dfrac{1}{a_2} - \dfrac{1}{a_3}\right) \\ B &= \rho g f_2\ \dfrac{V_1}{\pi a_2^2} - \rho g f_3\ \dfrac{V_1}{\pi a_3^2} - 4\sigma\left(\dfrac{1}{a_2} - \dfrac{1}{a_3}\right) \\ C &= -\dfrac{8\mu}{\pi^2 a_3^6}\ V_1 Q_1 - \rho g f_3\ \dfrac{V_1}{\pi a_3^2} - \dfrac{1}{2}\rho\left(\dfrac{Q_1}{\pi a_3^2}\right)^2 - 2\sigma\left(\dfrac{1}{a_2} - \dfrac{1}{a_3}\right) \end{cases} \tag{20}$$

3.2 Dimensionless equation

The quantities A, B, and C can be reformulated using the dimensionless numbers Re, Ca, and Bo:

$$Re = \frac{\rho U_1 a_1}{\mu} \quad , \quad Ca = \frac{\mu U_1}{\sigma} \quad , \quad Bo = \frac{\rho g a_1^2}{\sigma} \tag{21}$$

Then

$$\frac{8\mu}{\pi^2 a_2^6}\ V_1 Q_1 = \frac{8\mu}{\pi^2 a_2^6}\ \pi a_1^3 \left(\frac{V_1}{\pi a_1^3}\right) \pi a_1^2 U_1 = \frac{8\mu}{a_1 \lambda_2^6}\left(\frac{V_1}{\pi a_1^3}\right) U_1 = \frac{8\tilde{V}_1 Ca}{\lambda_2^6}\ \frac{\sigma}{a_1} \tag{22}$$

$$\rho g f_2\ \frac{V_1}{\pi a_2^2} = \frac{\sigma Bo}{a_1^2} f_2 \left(\frac{\pi a_1^3}{\pi a_2^2}\right) \tilde{V}_1 = \frac{f_2 \tilde{V}_1 Bo}{\lambda_2^2}\ \frac{\sigma}{a_1} \tag{23}$$

$$\frac{1}{2}\rho\left(\frac{Q_1}{\pi a_2^2}\right)^2 = \frac{1}{2}\rho \frac{\pi^2 a_1^4 U_1^2}{\pi^2 a_2^4} = \frac{ReCa}{2\lambda_2^4}\ \frac{\sigma}{a_1} \tag{24}$$

$$2\sigma\left(\frac{1}{a_2} - \frac{1}{a_3}\right) = 2\left(\frac{1}{\lambda_2} - \frac{1}{\lambda_3}\right)\ \frac{\sigma}{a_1} \tag{25}$$

The dimensionless equation on R_s is thus obtained by dividing all A, B, C coefficient by $\sigma/(2a_1)$:

$$
\begin{cases}
A' &= \dfrac{2a_1 A}{\sigma} = \dfrac{ReCa}{\lambda_2^4} + \dfrac{16\tilde{V}_1 Ca}{\lambda_2^6} + \dfrac{2\tilde{V}_1 Bo f_2}{\lambda_2^2} + 4\left(\dfrac{1}{\lambda_3} - \dfrac{1}{\lambda_2}\right) \\
B' &= \dfrac{2a_1 B}{\sigma} = 2\tilde{V}_1 Bo\left(\dfrac{f_2}{\lambda_2^2} - \dfrac{f_3}{\lambda_3^2}\right) + 8\left(\dfrac{1}{\lambda_3} - \dfrac{1}{\lambda_2}\right) \\
C' &= \dfrac{2a_1 C}{\sigma} = -\dfrac{ReCa}{\lambda_3^4} - \dfrac{16\tilde{V}_1 Ca}{\lambda_3^6} - \dfrac{2\tilde{V}_1 Bo f_3}{\lambda_3^2} + 4\left(\dfrac{1}{\lambda_3} - \dfrac{1}{\lambda_2}\right)
\end{cases}
\tag{26}
$$

3.3 The splitting factor

The splitting ratio ranges from 0 to infinity, and can become infinite when the entire plug goes to the daughter branch number 2. To avoid handling infinite quantities, one introduces instead the *splitting factor* α defined as:

$$
\alpha = \frac{R_s}{1+R_s} \qquad \text{which means that} \quad R_s = \frac{\alpha}{1-\alpha} \tag{27}
$$

α ranges from 0 to 1. It is 0 when all the liquid goes to daughter branch number 3, and is 1 when all the liquid goes to daughter branch number 2. α satisfies a second order equation which is obtained from the dimensionless equation in R_s:

$$
A'\left(\frac{\alpha}{1-\alpha}\right)^2 + B'\frac{\alpha}{1-\alpha} + C' = 0 \tag{28}
$$

hence

$$
A_1\alpha^2 + B_1\alpha + C_1 = 0 \qquad \text{with} \quad
\begin{cases}
A_1 &= A' - B' + C' \\
B_1 &= B' - 2C' \\
C_1 &= C'
\end{cases}
\tag{29}
$$

Consequently, the coefficients A_1, B_1, and C_1 are:

$$
\begin{cases}
A_1 &= ReCa\left(\dfrac{1}{\lambda_2^4} - \dfrac{1}{\lambda_3^4}\right) + 16\tilde{V}_1 Ca\left(\dfrac{1}{\lambda_2^6} - \dfrac{1}{\lambda_3^6}\right) \\
B_1 &= 2\tilde{V}_1 Bo\left(\dfrac{f_2}{\lambda_2^2} + \dfrac{f_3}{\lambda_3^2}\right) + \dfrac{2ReCa}{\lambda_3^4} + \dfrac{32\tilde{V}_1 Ca}{\lambda_3^6} \\
C_1 &= -\dfrac{ReCa}{\lambda_3^4} - \dfrac{16\tilde{V}_1 Ca}{\lambda_3^6} - \dfrac{2\tilde{V}_1 Bo f_3}{\lambda_3^2} + 4\left(\dfrac{1}{\lambda_3} - \dfrac{1}{\lambda_2}\right)
\end{cases}
\tag{30}
$$

3.4 The symmetric bifurcation

In a symmetric bifurcation, $\lambda_2 = \lambda_3 = \lambda$ and $\theta_3 = -\theta_2$. This simplifies the expression of A_1, B_1, and C_1 to:

$$\begin{cases} A_1 & = 0 \\ B_1 & = \dfrac{2\tilde{V}_1 Bo(f_2 + f_3)}{\lambda^2} + \dfrac{2ReCa}{\lambda^4} + \dfrac{32\tilde{V}_1 Ca}{\lambda^6} \\ C_1 & = -\dfrac{2\tilde{V}_1 Bo f_3}{\lambda^2} - \dfrac{ReCa}{\lambda^4} - \dfrac{16\tilde{V}_1 Ca}{\lambda^6} \end{cases} \tag{31}$$

The equation in α is now a first order equation whose solution is

$$\begin{aligned} \alpha &= \frac{\dfrac{2\tilde{V}_1 Bo f_3}{\lambda^2} + \dfrac{ReCa}{\lambda^4} + \dfrac{16\tilde{V}_1 Ca}{\lambda^6}}{\dfrac{2\tilde{V}_1 Bo(f_2 + f_3)}{\lambda^2} + \dfrac{2ReCa}{\lambda^4} + \dfrac{32\tilde{V}_1 Ca}{\lambda^6}} \\ &= \frac{2\tilde{V}_1 Bo f_3 \lambda^4 + ReCa\lambda^2 + 16\tilde{V}_1 Ca}{2\tilde{V}_1 Bo(f_2 + f_3)\lambda^4 + 2ReCa\lambda^2 + 32\tilde{V}_1 Ca} \end{aligned} \tag{32}$$

One can introduce the dimensionless quantity X defined as:

$$X = \frac{2B_0 \tilde{V}_1 \lambda^4}{\left(Re\lambda^2 + 16\tilde{V}_1\right) Ca}. \tag{33}$$

Using it, one finally expresses the splitting factor α:

$$\alpha = \frac{1 + X f_3}{2 + X(f_2 + f_3)} = \frac{1}{2} + \frac{X(f_3 - f_2)}{4 + 2X(f_2 + f_3)}, \tag{34}$$

$f_2 + f_3$ and $f_3 - f_2$ being now:

$$f_2 + f_3 = -2\cos\theta \sin\gamma \qquad \text{and} \qquad f_3 - f_2 = -2\sin\theta\sin\varphi \tag{35}$$

which finally gives:

$$\alpha = \frac{1}{2}\left(1 - \frac{X\sin\theta\sin\varphi}{1 - X\cos\theta\sin\gamma}\right) \tag{36}$$
